# *Anaplasma phagocytophilum* Ats-1 Is Imported into Host Cell Mitochondria and Interferes with Apoptosis Induction

**DOI:** 10.1371/journal.ppat.1000774

**Published:** 2010-02-19

**Authors:** Hua Niu, Vera Kozjak-Pavlovic, Thomas Rudel, Yasuko Rikihisa

**Affiliations:** 1 Department of Veterinary Biosciences, The Ohio State University, Columbus, Ohio, United States of America; 2 Biocenter, Department of Microbiology, University of Würzburg, Am Hubland, Würzburg, Germany; Duke University, United States of America

## Abstract

*Anaplasma phagocytophilum,* the causative agent of human granulocytic anaplasmosis, infects human neutrophils and inhibits mitochondria-mediated apoptosis. Bacterial factors involved in this process are unknown. In the present study, we screened a genomic DNA library of *A. phagocytophilum* for effectors of the type IV secretion system by a bacterial two-hybrid system, using *A. phagocytophilum* VirD4 as bait. A hypothetical protein was identified as a putative effector, hereby named *Anaplasma* translocated substrate 1 (Ats-1). Using triple immunofluorescence labeling and Western blot analysis of infected cells, including human neutrophils, we determined that Ats-1 is abundantly expressed by *A. phagocytophilum*, translocated across the inclusion membrane, localized in the host cell mitochondria, and cleaved. Ectopically expressed Ats-1 targeted mitochondria in an N-terminal 17 residue-dependent manner, localized in matrix or at the inner membrane, and was cleaved as native protein, which required residues 55–57. *In vitro*-translated Ats-1 was imported in a receptor-dependent manner into isolated mitochondria. Ats-1 inhibited etoposide-induced cytochrome *c* release from mitochondria, PARP cleavage, and apoptosis in mammalian cells, as well as Bax-induced yeast apoptosis. Ats-1(55–57) had significantly reduced anti-apoptotic activity. Bax redistribution was inhibited in both etoposide-induced and Bax-induced apoptosis by Ats-1. Taken together, Ats-1 is the first example of a bacterial protein that traverses five membranes and prevents apoptosis at the mitochondria.

## Author Summary

*Anaplasma phagocytophilum* is the pathogen that causes human granulocytic anaplasmosis, an emerging infectious disease. As an obligate intracellular organism, this bacterium cannot reproduce outside of eukaryotic cells due to the loss of many genes that are present in free-living bacteria. Paradoxically, it specifically infects short-lived white blood cells that play critical roles in anti-microbial defense, by subverting a number of host innate immune responses including programmed cell death (apoptosis). *A. phagocytophilum* factors that are involved in this process are largely unknown. In this study, we first searched *A. phagocytophilum* proteins that are secreted by its specialized secretion system into eukaryotic cells. We found a protein of unknown function, here named Ats-1, which is abundantly produced by *A. phagocytophilum* and traverses five membranes to enter the mitochondria of human cells. Our further study showed that Ats-1 reduces the sensitivity of mitochondria to respond to apoptosis-inducing factors, leading to the inhibition of host cell apoptosis. Thus, present findings identified a bacterial protein that allows infected white blood cells to live longer to support bacterial growth. The absence of similarity of the sequence or the mode of action to any other known cell death suppressor suggests that Ats-1 defines a previously undescribed class of anti-apoptotic protein. This protein and the mechanism thereof may provide insight regarding a new therapeutic target for treatment of human granulocytic anaplasmosis.

## Introduction

Infection of humans with rickettsia, *Anaplasma phagocytophilum* leads to an acute febrile systemic disease called human granulocytic anaplasmosis, which is classified as an emerging infectious disease. Immune-compromised, elderly, or individuals burdened with preexisting health conditions are at high risk for severe complications that can result in death [1],[2]. *A. phagocytophilum* is an obligatory intracellular bacterium that cannot reproduce outside of eukaryotic cells due to the loss of many genes present in free-living bacteria [3],[4]. Paradoxically, this is also one of few bacteria that are capable of specifically infecting short-lived neutrophils, which are equipped with powerful anti-microbial defense. It is noteworthy that *A. phagocytophilum* subverts a number of host innate immune responses including programmed cell death (apoptosis) [5].

Apoptosis of infected cells is one of the important innate immune responses against intracellular pathogens, including viruses, bacteria, and parasites [6]. Neutrophils typically undergo spontaneous apoptosis within 6–12 h after release into the peripheral blood from the bone marrow, an important process in the maintenance of homeostatic levels of neutrophils and in the resolution of inflammatory responses [7]. *A. phagocytophilum* infection inhibits spontaneous and induced apoptosis of isolated peripheral blood human neutrophils for up to 48 h and of neutrophils in peripheral blood leukocyte cultures for up to 96 h as determined by morphological observation [8]. This *A. phagocytophilum* anti-apoptotic phenomenon has been confirmed by several *in vitro* studies on human neutrophils as well as by an *ex vivo* study on ovine neutrophils infected *in vivo* with a sheep isolate [9],[10],[11],[12],[13],[14]. This delay of neutrophil apoptosis allows sufficient time for the intracellular replication of the bacteria [8]. The cellular mechanisms by which *A. phagocytophilum* inhibits the apoptosis of human neutrophils include inhibition of the loss of mitochondrial membrane potential, Bax translocation to the mitochondria, and the activation of downstream caspase 3 [12],[13]. However, bacterial factors involved in these processes are unknown.

Evolved from the bacterial conjugation system, the type IV secretion (T4S) system transports macromolecules across the bacterial membrane in an ATP-dependent manner into a diverse range of eukaryotic cells [15]. The T4S system has been recognized as the machinery for virulence factor delivery of host cell-associated bacterial pathogens. The delivered bacterial macromolecules referred to as T4S substrates or effectors can dysregulate or modulate diverse eukaryotic target cell functions, resulting in disease development [15]. It is important to note that three T4S substrates, BepA of *Bartonella henselae,* and SdhA and SidF of *Legionella pneumophila* are known to be involved in host cell apoptosis [16],[17],[18], although where these proteins are localized in infected cells is unknown.

Encoding components of the T4S apparatus, *Agrobacterium tumefaciens virB/virD* homologs have been identified in *A. phagocytophilum* [4],[19]. During the infection of human neutrophils *in vitro*, *virB9* and *virB6*, two T4S apparatus protein mRNAs and VirB9 protein are up-regulated [20]. Although targeted manipulation of genes currently is not applicable to obligatory intracellular bacteria, VirD4-dependent secretion of the *A. phagocytophilum* ankyrin repeat protein, AnkA, has been demonstrated using the *A. tumefaciens* Cre recombinase reporter assay for translocation [21].

*A. tumefaciens* VirD4, a component of the T4S apparatus localized in the cytoplasmic membrane, is regarded as a coupling protein, because it recognizes C-terminal sequences within T4S substrate proteins prior to delivery into the VirB transmembrane channel [22]. In the present study, we screened an *A. phagocytophilum* genomic DNA library for T4S substrates by bacterial two-hybrid system using *A. phagocytophilum* VirD4 (GenBank YP_505894) as bait. A hypothetical protein was identified as a putative substrate, hereby named *Anaplasma* translocated substrate 1 (Ats-1). Our results demonstrate that Ats-1 is translocated across the bacterial and inclusion membranes, localized in the mitochondrial matrix, or at inner membrane, and cleaved in the infected cells. Given the distinct localization in the mitochondria, the integrators of pro-and anti-apoptotic signaling, we also address the possible role of Ats-1 in inhibition of mitochondria-mediated host cell apoptosis. Our data suggest that Ats-1 reduces the sensitivity of host cell mitochondria to apoptosis-inducing stimulus by inhibiting Bax redistribution to the mitochondria.

## Results

### Identification of VirD4-interacting proteins

To identify potential T4S effectors/substrates, we screened an *A. phagocytophilum* genomic DNA library by bacterial two-hybrid system using full-length *A. phagocytophilum* VirD4 as bait. One hundred bacterial colonies were sequenced, the majority of which (65%) encoded amino acid residues 9–253 of a 253-residue hypothetical protein APH0859 (GenBank YP_505436). Eight colonies encoded residues 210–332 of the 332-residue protein VirB11 (GenBank YP_505895). The remaining 27 colonies encoded various *A. phagocytophilum* proteins, each with a 1- or 2-hit frequency. No colonies grew in selective media when empty bait vector pBT and prey vector pTRG-APH0859 isolated from bacterial two-hybrid screening were used to co-transform the *E. coli* reporter strain, indicating that the interaction between VirD4 and APH0859 was specific (data not shown). This result indicates that APH0859 is a potential substrate for the T4S system of *A. phagocytophilum.* APH0859 is annotated in GenBank as a protein with a predicted molecular mass of 27 kDa and a predicted pI of 6.64 [4]. The C-terminus of APH0859 (20 residues: VTPLVSAQNRGPETHGKGTR) bears more basic amino acids than the remainder of the protein, which is similar to *A. tumefaciens* T4S substrates [23]. These 20 residues have a net positive charge of +2.077 at pH 7.0, with a calculated pI of 10.89. The direct interaction between the two inner membrane ATPases VirD4 and VirB11 of *A. phagocytophilum* affirms the previously indicated VirD4-VirB11 interaction in *A. tumefaciens*, which was shown by genetic suppression, transfer DNA immunoprecipitation, and coimmunoprecipitation experiments [24],[25]. The interaction between *A. phagocytophilum* VirD4 and *A. phagocytophilum* VirB11 suggests that *A. phagocytophilum* VirD4 may usher T4S substrates to VirB11 prior to delivery to the core T4S channel, as demonstrated in *A. tumefaciens* for delivery of T-DNA [26].

### Secretion of *A. phagocytophilum* APH0859

**Figure 1:**
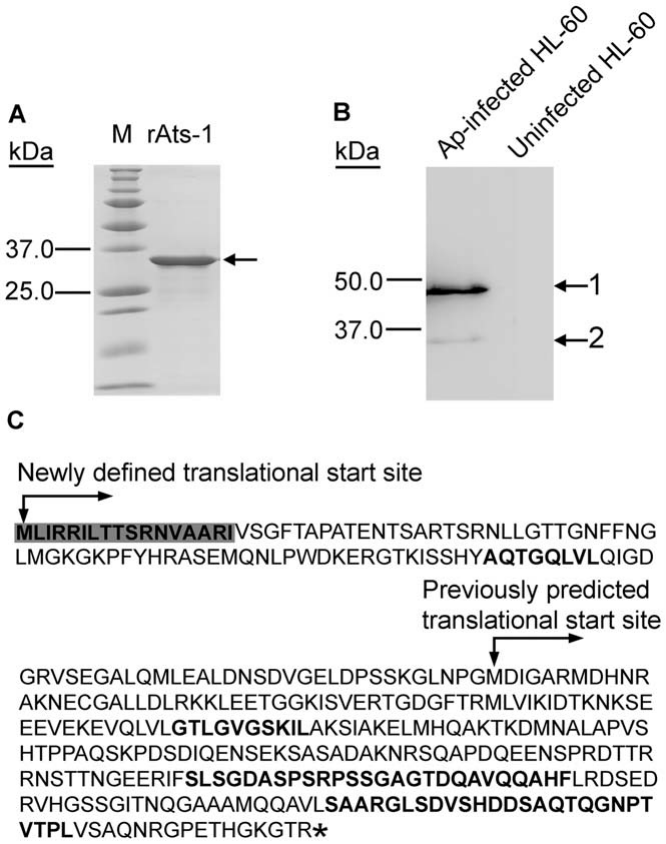
Expression of APH0859 in *Anaplasma phagocytophilum* and new annotation of *aph0859* ORF. **A.** Truncated rAts-1 (derived from predicted APH0859 ORF) expressed in *E. coli* was purified by immobilized Ni^2+^ affinity chromatography, and subjected to SDS-PAGE analysis followed by Coomassie Brilliant blue staining. Lanes: M, protein molecular mass marker; rAts-1, recombinant truncated *A. phagocytophilum* Ats-1. Arrow indicates rAts-1 migration. **B.** Western blot analysis of *A. phagocytophilum* (Ap)-infected and uninfected HL-60 cells using affinity-purified rabbit anti-Ats-1 antibody. Arrows 1 and 2 indicate full-length native Ats-1 and cleaved Ats-1, respectively. Molecular mass markers are indicated at the left. **C.** The amino acid sequence deduced from the newly defined *aph0859* (*ats-1*) ORF. The amino acid sequences identified by mass spectrometry are highlighted in bold. The N-terminal mitochondrial targeting sequence predicted by the Mitoprot program is indicated in shaded bold. * indicates the stop codon.

If APH0859 is the true substrate of the T4S system, it is secreted from the bacteria. In order to determine protein expression and secretion, the gene *aph0859,* encoding the 253-residue protein, was cloned into pET-33b(+) and expressed as a recombinant protein in *E. coli*. The purified recombinant protein (Figure 1A) was used to immunize rabbits. An antibody with monospecificity to recombinant APH0859 (rAPH0859) was affinity-purified using rAPH0859-conjugated Affi-gel 10 agarose. Using the anti-rAPH0859 antibody, Western blot analysis revealed one highly immunoreactive protein of approximately 48 kDa, and one weakly immunoreactive protein of approximately 35 kDa in *A. phagocytophilum*-infected HL-60 cells, a human promyelocytic leukemia cell line, whereas no immunoreactive proteins were detected in uninfected HL-60 cells (Figure 1B). Since the molecular mass of APH0859 in the Western blot analysis was larger than the expected molecular mass (27 kDa), the open reading frame (ORF) of *aph0859* was reanalyzed. Consequently, a new ORF for *aph0859* (GenBank FJ210653) was defined that encoded a protein of 376 residues (calculated molecular mass: 40.3 kDa); the most upstream ATG was designated as the translational start site. The new annotation of this ORF was verified by several methods. First, when the 48-kDa protein was immunoprecipitated from *A. phagocytophilum*-infected HL-60 cell lysate using anti-rAPH0859 antibody, and subjected to protein identification by mass spectrometry, a peptide with the sequence matching only the N-terminus of the newly defined APH0859 ORF was identified, in addition to several peptides matching the previously annotated APH0859 (Figure 1C). Second, recombinant expression of the new ORF in *E. coli* resulted in synthesis of a polypeptide with a molecular mass similar to that of the native APH0859 protein (data not shown). Third, when the new ORF sequence was aligned with the *Anaplasma marginale* ortholog AM410 (predicted molecular mass 43 kDa, GenBank YP_153722), the N-terminal region of the newly defined APH0859 (residues 1–123) was similar to that of AM410 (E value = 1.2e–09). The 48-kDa protein encoded by this newly defined *aph0859* ORF was named *Anaplasma* translocation substrate 1 (Ats-1). Although the basis for the discrepancy between the calculated molecular mass of Ats-1 (40.3 kDa) and the mass (48 kDa) visualized by SDS-PAGE remains elusive, such discrepancies have been observed for other *Anaplasma* proteins such as AnkA [21].

**Figure 2:**
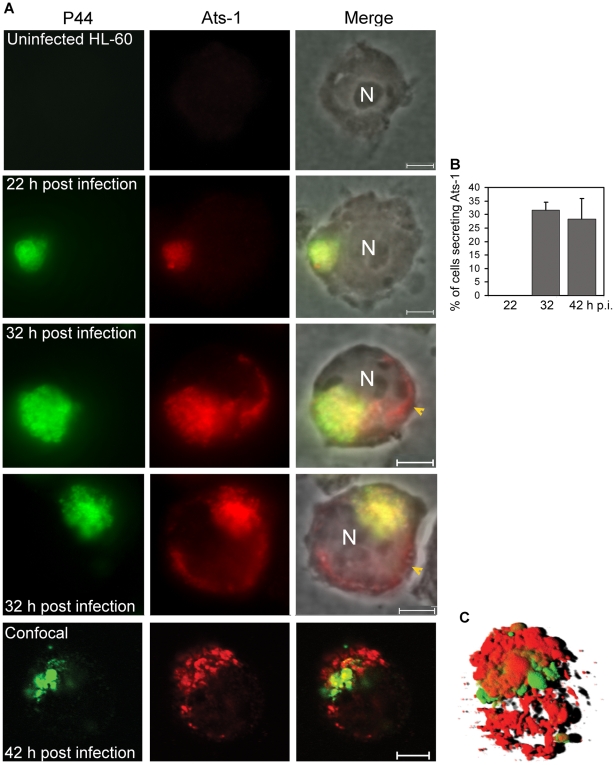
Translocation of Ats-1 from *Anaplasma phagocytophilum* into the cytoplasm of HL-60 cells. **A.** Double immunofluorescence labeling of Ats-1 and P44 in *A. phagocytophilum*–infected HL-60 cells. Uninfected HL-60 cells and *A. phagocytophilum*-infected HL-60 cells at 22, 32, or 42 h post-infection were double immunofluorescence-labeled using mouse monoclonal anti-P44 (P44; Alexa Fluor 488, green) and affinity-purified rabbit anti-Ats-1 (Ats-1; Alexa Fluor 555, red), and subjected to epifluorescence microscopy (22 or 32 h p.i.) or to confocal laser scanning microscopy (42 h p.i.). Merged images with or without phase contrast micrograph are shown (Merge). N: nucleus. Yellow arrowheads indicate Ats-1 translocated to the cytoplasm of host cells. Scale bar: 5 µm. **B.** Percentage of HL-60 cells with P44 signal having non-colocalized (secreted) Ats-1 signal at 22, 32 or 42 h p.i. One hundred *A. phagocytophilum* P44-positive HL-60 cells at 22, 32 or 42 h p.i. were scored, and the percentage of cells which has Ats-1 cytoplasmic signal was determined. Data are presented as the means and standard deviations of triplicate samples. **C.** A 3-D shadow projection image was reconstructed based on the Z-stack data from confocal microscopy, performed for *A. phagocytophilum*-infected HL-60 cells at 42 h p.i. Green color: P44; Red color: Ats-1.

To determine whether Ats-1 is expressed by *A. phagocytophilum* and secreted into the host cell cytoplasm across the inclusion and bacterial membranes, double immunofluorescence labeling was performed. Since P44 is the major outer membrane protein of *A. phagocytophilum* [27], monoclonal anti-P44 antibody 5C11 [28] was used to label *A. phagocytophilum*, and rabbit anti-Ats-1 antibody was used to localize Ats-1 following *A. phagocytophilum* infection of HL-60 cells. Ats-1 was expressed and colocalized with *A. phagocytophilum* at 22 h post-infection (p.i.) by double immunofluorescence labeling. Ats-1 secretion into the host cell cytoplasm became obvious after 32 h p.i. as the lack of P44 colocalization with a portion of Ats-1 (Figure 2A). At 32 h and 42 h p.i, the percentage of infected cells that secreted the Ats-1 signal was approximately 32 and 28%, respectively (Figure 2B). The 3-D shadow projection image constructed based on the Z-stack data from confocal microscopy, affirmed that Ats-1 was secreted into host cytoplasm from *A. phagocytophilum,* and some of the protein remained externally associated with the bacteria or bacterial inclusions (Figure 2C). In addition, a 3-D reconstruction video is shown in the supplementary data (Video S1).

### Ats-1 targets host cell mitochondria in an N-terminal sequence-dependent manner

**Figure 3:**
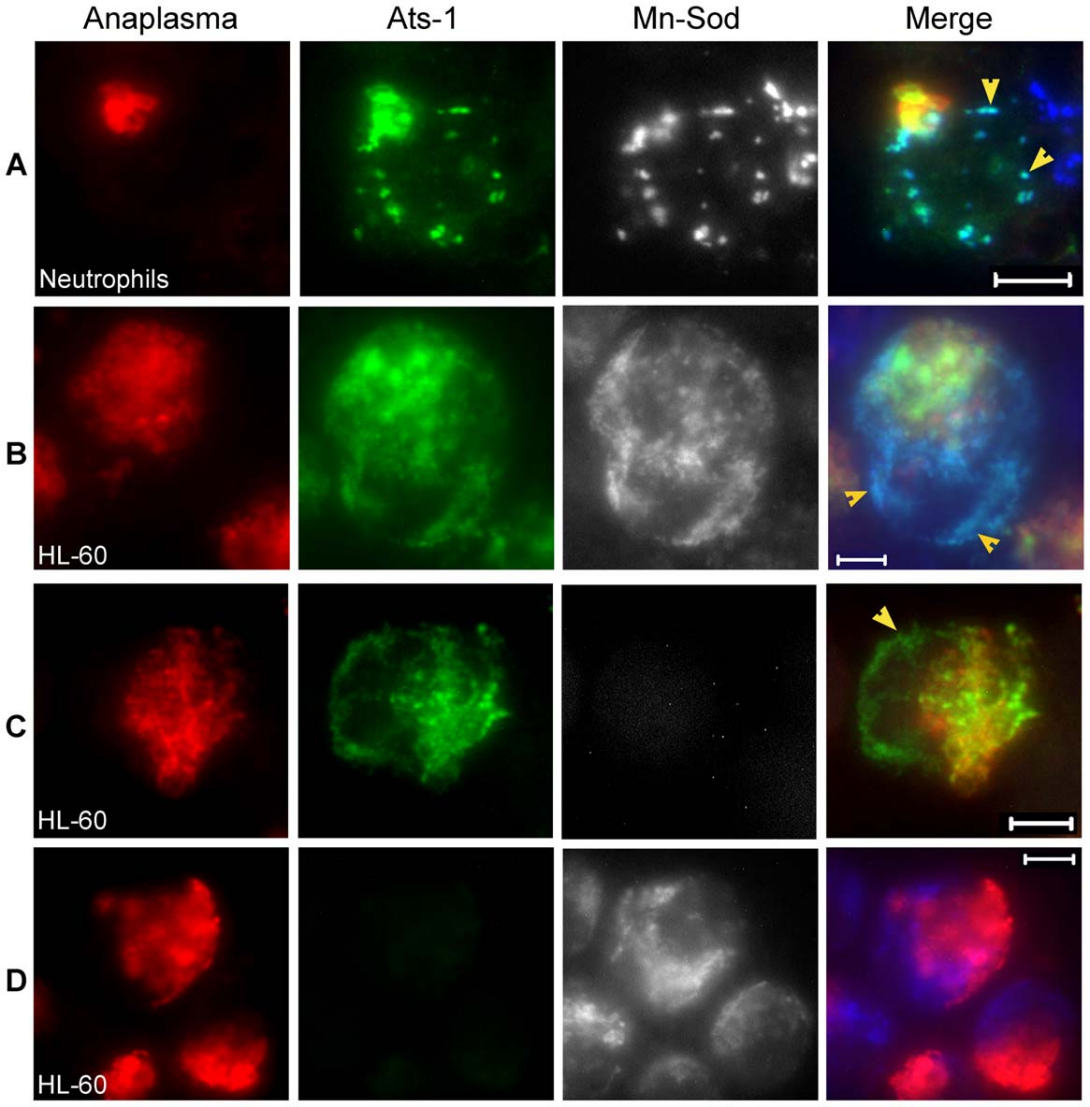
Ats-1 localizes with mitochondria in host cells. **A and B.** Triple immunofluorescence labeling of *A. phagocytophilum-*infected human neutrophils (A) and HL-60 cells (B) using horse anti-*A. phagocytophilum* (Anaplasma; Cy3, red), rabbit anti-Ats-1 (Ats-1; Alexa Fluor 488, green) and monoclonal anti-Mn-Sod (Mn-Sod; Alexa Fluor 350, gray pseudocolor). Arrowheads indicate Ats-1 and mitochondria colocalization. Scale bar: 5 µm. **C and D.** Negative controls for Ats-1 or Mn-Sod staining in *A. phagocytophilum*–infected HL-60. *A. phagocytophilum*–infected HL-60 cells were stained with primary antibodies, horse anti-*A. phagocytophilum,* and rabbit anti-Ats-1 (C), or monoclonal anti-Mn-Sod (D), then stained with secondary antibodies, Cy3-conjugated goat anti-horse IgG, Alexa Fluor 488-conjugated goat anti-rabbit IgG, and Alexa Fluor 350-conjugated goat anti-mouse IgG (gray pseudocolor). Arrowhead indicates secreted Ats-1. Scale bar: 5 µm.

The distribution of secreted Ats-1 in HL-60 cells was not homogenous, but granular (Figures 2A and 2C). In addition, we identified a mitochondrial localization signal at the N-terminus of Ats-1 based on two *in silico* prediction programs: MitoProt (http://ihg.gsf.de/ihg/mitoprot.html) and Predotar (http://urgi.versailles.inra.fr/predotar/predotar.html) (Figure 1C) [29],[30]. Therefore, we examined whether secreted Ats-1 targets the mitochondria in *A. phagocytophilum*-infected human neutrophils (natural host cells) and HL-60 cells by triple immunofluorescence labeling. Horse anti-*A. phagocytophilum* serum and Cy3-conjugated goat anti-horse IgG were used to label *A. phagocytophilum*; rabbit anti-Ats-1 antibody and Alexa Fluor 488-conjugated goat anti-rabbit IgG were used for Ats-1 labeling; mouse monoclonal anti-manganese superoxide dismutase (Mn-Sod), a mitochondrial marker, and Alexa Fluor 350-conjugated goat anti-mouse IgG were used for mitochondria labeling as reported previously [13]. Some Ats-1 localized with *A. phagocytophilum*, and the rest of Ats-1 that did not colocalize with *A. phagocytophilum*, colocalized with mitochondria in infected human neutrophils and HL-60 cells (Figures 3A and 3B). This result suggests that Ats-1 targets mitochondria after secretion into the host cell cytoplasm. There was no cross-reaction or bleed-through between the Ats-1 signal and Mn-Sod signal, as indicated from the negative controls (Figures 3C and 3D).

**Figure 4:**
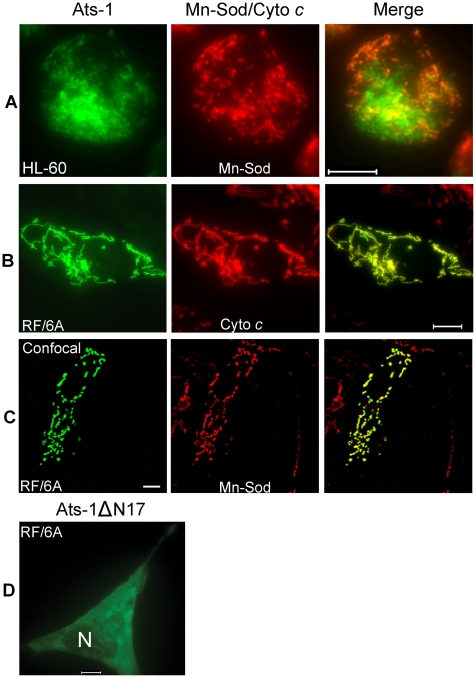
Mitochondrial targeting of Ats-1, and essential role of N-terminal sequence in targeting. **A-C.** Double immunofluorescence labeling of *A. phagocytophilum*–infected HL-60 cells (A), or pAts-1-transfected RF/6A cells (B and C) using rabbit anti-Ats-1 (Ats-1; Alexa Fluor 488, green), and monoclonal anti-Mn-Sod (Mn-Sod; Alexa Fluor 555, red) (A and C), monoclonal anti-cytochrome *c* (Cyto *c*; Alexa Fluor 555, red) (B). Scale bar: 10 µm. **D.** Immunofluorescence labeling of RF/6A cells transfected with pAts-1ΔN17 was performed using anti–Ats-1 (Alexa Fluor 488, green). N, nucleus. Note diffuse distribution of Ats-1ΔN17 in the cytoplasm of RF/6A cell. Scale bar: 10 µm.

To determine whether translocation to the mitochondria is an intrinsic property of Ats-1, *ats-1* was ectopically expressed in RF/6A monkey endothelial cells. No tag was added to Ats-1 to prevent mis-targeting, as it was found that GFP tag alters Ats-1 cellular localization (Data not shown). *A. phagocytophilum* was shown previously to infect endothelial cells *in vitro* and *in vivo* [31],[32]. In addition, RF/6A cells are more easily transfected than HL-60 cells, and the mitochondria can be more easily recognized under a fluorescence microscope due to the characteristic filamentous and flat shape of the cells (Figures 4B and 4C), whereas in neutrophils and HL-60 cells, mitochondria and cells are round making them difficult to focus simultaneously (Figures 3A, 3B, and 4A). Using double immunofluorescence labeling, we determined that Ats-1 was expressed in pAts-1-transfected RF/6A cells and colocalized with cytochrome *c* and Mn-Sod, indicating Ats-1 is translocated to the mitochondria in the absence of any other bacterial factors (Figures 4B and 4C). Ats-1 with the putative N-terminal mitochondrial targeting sequence deletion (Ats-1ΔN17) showed the diffuse distribution in RF/6A cells, indicating N17 of Ats-1 is required for mitochondrial targeting (Figure 4D).

### Ats-1 is cleaved in the mitochondria

**Figure 5:**
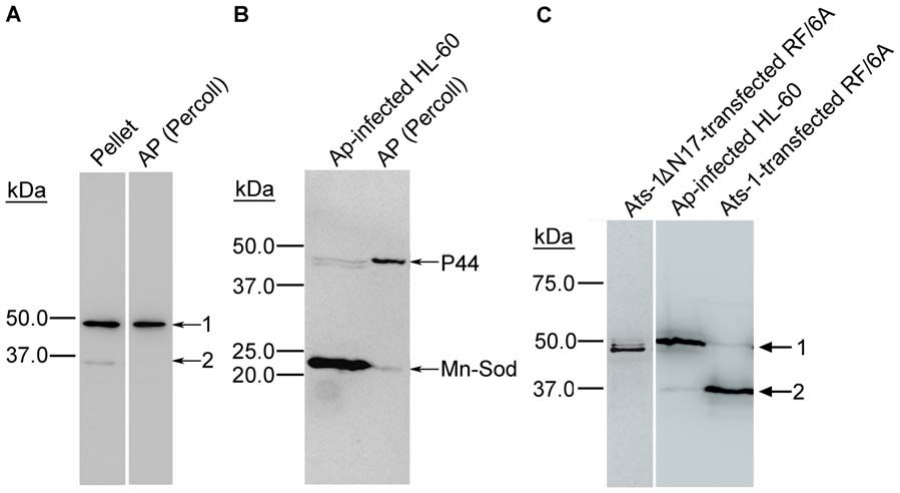
Ats-1 is cleaved in the mitochondria. **A.** Western blot analysis of the *A. phagocytophilum*- and mitochondria-containing pellet (Pellet) after 10,000×g centrifugation, and *A. phagocytophilum* purified from the pellet by Percoll density-gradient centrifugation [AP (Percoll)] using anti-Ats-1. Arrows 1 and 2 indicate full-length native Ats-1 and cleaved Ats-1, respectively. Molecular mass markers are indicated at left. **B.** Western blot analysis of *A. phagocytophilum* (Ap)-infected HL-60 cells and percoll-purified *A. phagocytophilum* [AP (Percoll)], using mouse monoclonal anti-P44 and rabbit anti-Mn-Sod. Molecular mass markers are indicated at left. **C.** Western blot analysis of pAts-1 or pAts-1ΔN17-transfected RF/6A cells using anti-Ats-1. Ats-1 was cleaved in *A. phagocytophilum* (Ap)-infected HL-60 cells and pAts-1-transfected cells, but not in pAts-1ΔN17-transfected cells. Arrows 1 and 2 indicate full-length Ats-1 and cleaved Ats-1, respectively. Molecular mass markers are indicated at left.

Most of mitochondrial proteins are encoded by nuclear DNA in eukaryotes, and many of them are translated as precursor proteins, from which the N-terminal presequence including the mitochondrial targeting sequence is cleaved by the matrix-located mitochondrial processing peptidase [33]. Although, there has been no example of any native bacterial protein processed by the mitochondrial matrix peptidase, we wondered whether native Ats-1 is cleaved in the mitochondria, as two Ats-1 species (35 kDa and 48 kDa) were detected in *A. phagocytophilum-*infected HL-60 cells by Western blot analysis (Figure 1B). The 48- and 35-kDa Ats-1s were detected in the *A. phagocytophilum* and mitochondria pellet obtained by 10,000×g centrifugation of the infected cell lysate (Figure 5A), whereas only a single 48-kDa Ats-1 species was detected in *A. phagocytophilum* isolated from the 10,000×g pellet by Percoll density-gradient centrifugation (Figure 5A). Mitochondria (Mn-Sod as marker) were mostly removed from Percoll density-gradient purified *A. phagocytophilum* (Figure 5B). This result suggests that native Ats-1 (48 kDa) was cleaved to 35 kDa after translocation to the host cell mitochondria. In agreement with the results of colocalization of almost all Ats-1 with mitochondria, when Ats-1 was ectopically expressed in RF/6A cells (Figures 4B and 4C), the 35-kDa species represented the major form (Figure 5C). This result also confirms that Ats-1 was cleaved by the host cell protease. Ats-1ΔN17 ectopically expressed in RF/6A cells, was not cleaved, indicating Ats-1 mitochondrial localization is required for the cleavage (Figure 5C).

### Ats-1 is a precursor protein and the presequence is cleaved at a specific site

**Figure 6:**
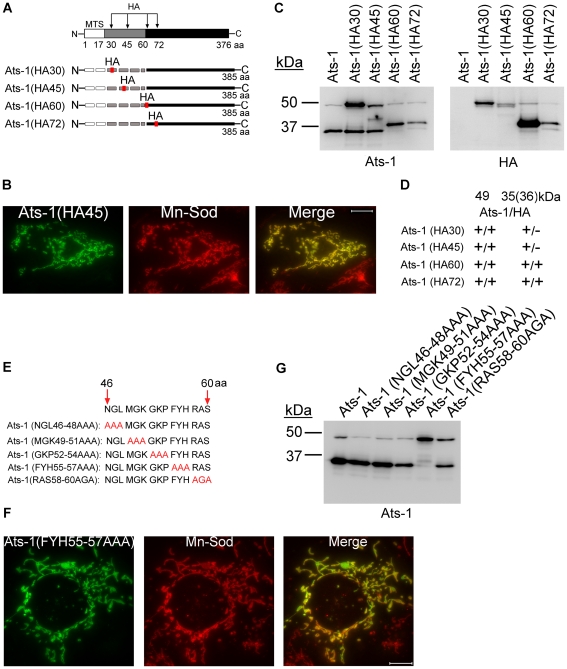
Ats-1 presequence is cleaved at a specific site. **A.** Schematic diagram indicating HA tag insertion and predicted cleavage sites in Ats-1 mutants. The HA tag was inserted between residues 30 and 31, 45 and 46, 60 and 61, or 72 and 73 of Ats-1. MTS, predicted mitochondrial targeting sequence, indicated by white bar. Black bar indicates cleaved Ats-1 fragments detected by Western blot analysis; Gray bar indicates the sequence between MTS and cleavage site. Dashed lines indicate undetectable (degraded) N-terminal cleaved fragment. **B.** Immunofluorescence labeling of RF/6A cells transfected with pAts-1 (HA45) using rabbit anti-Ats-1 [Ats-1(HA45); Alexa Fluor 488, green] and mouse monoclonal anti-Mn-Sod (Mn-Sod; Alexa Fluor 555, red). Note mitochondrial localization of Ats-1 (yellow). Scale bar: 10 µm. **C.** Western blot analysis using anti-Ats-1 or monoclonal anti-HA antibody was performed to examine Ats-1 cleavage in RF/6A cells transfected with recombinant plasmids encoding wild type Ats-1 or Ats-1 mutants with HA insertion at different locations. Molecular mass markers are indicated at left. **D.** Summarized result for Figure 6C. The molecular size for four Ats-1 mutants in Western blot analysis using anti-Ats-1 (Ats-1) or anti-HA (HA) antibody for Ats-1 (49 kDa) and cleaved Ats-1 (35, or 36 kDa). + indicates immunoreactivity; - indicates no immunoreactivity. Note the molecular mass of full-length Ats-1 or cleaved Ats-1 which has HA insertion increases 1 kDa. **E.** Diagram showing sequential substitution of amino acid triplets within residues 46–60 of full-length Ats-1. The wild type Ats-1 sequence at residues 46–60 was shown at the top and the mutant sequences in this region are shown below. Substituted residues were indicated in red. **F.** Immunofluorescence labeling of RF/6A cells transfected with pAts-1 (FYH55-57AAA) using rabbit anti-Ats-1 [Ats-1(FYH55-57AAA); Alexa Fluor 488, green] and mouse monoclonal anti–Mn-Sod (Mn-Sod; Alexa Fluor 555, red). Note mitochondrial localization of Ats-1 (yellow). Scale bar: 10 µm. **G.** Western blot analysis using anti–Ats-1 was performed to examine Ats-1 cleavage in RF/6A cells transfected with recombinant plasmids encoding wild type Ats-1 or the indicated Ats-1 triplet substitution mutant. Cleavage was inhibited only in cells expressing the Ats-1 (FYH55-57AAA) mutant. Molecular mass markers are indicated at left.

To determine whether the N-terminal presequence is cleaved from the precursor 48-kDa Ats-1 to generate the mature 35-kDa Ats-1 in the mitochondria, a sequence encoding a hemagglutinin (HA) tag (YPYDVPDYA) was inserted in-frame into the Ats-1 expression plasmid at different locations to create plasmids expressing Ats-1 (HA30), Ats-1 (HA45), Ats-1 (HA60), or Ats-1 (HA72) (Figure 6A). The mutant proteins were ectopically expressed in RF/6A cells, and their mitochondrial localization and cleavage were examined. All four Ats-1 mutants colocalized with mitochondria [Figure 6B, only Ats-1 (HA45) is shown] and cleaved, generating ∼35-kDa species (Figure 6C). Thus, HA tag insertion at these locations did not prevent Ats-1 mitochondrial targeting. However, protease sensitivity of Ats-1 (HA30) and Ats-1 (HA45) was reduced compared to wild-type Ats-1, Ats-1 (HA60), and Ats-1 (HA72), indicating that former insertions influence Ats-1 sub-mitochondrial processing. The ∼35-kDa cleaved products of Ats-1 (HA30) and Ats-1 (HA45) did not have the HA tag, but the cleaved products of Ats-1 (HA60) and Ats-1 (HA72) did (Figures 6C and 6D). The result indicates that the cleavage site of Ats-1 is within N-terminal residues 45–60. To determine which residues within Ats-1 residues 45–60 are important for cleavage, the amino acids within this region were sequentially substituted with AAA or AGA (Figure 6E). All five mutants colocalized with mitochondria (Figure 6F, only Ats-1 (FYH55-57AAA) mutant [Ats-1(55–57)] is shown). However, of the five mutants, substantial cleavage inhibition was observed only for Ats-1(55–57) (Figure 6G). These results indicate 48-kDa Ats-1 is a precursor to the 35-kDa mature protein and that residues 55–57 of Ats-1 are critical for processing to generate the mature Ats-1.

### Ats-1 is routed to the mitochondrial matrix in receptor-dependent manner

**Figure 7:**
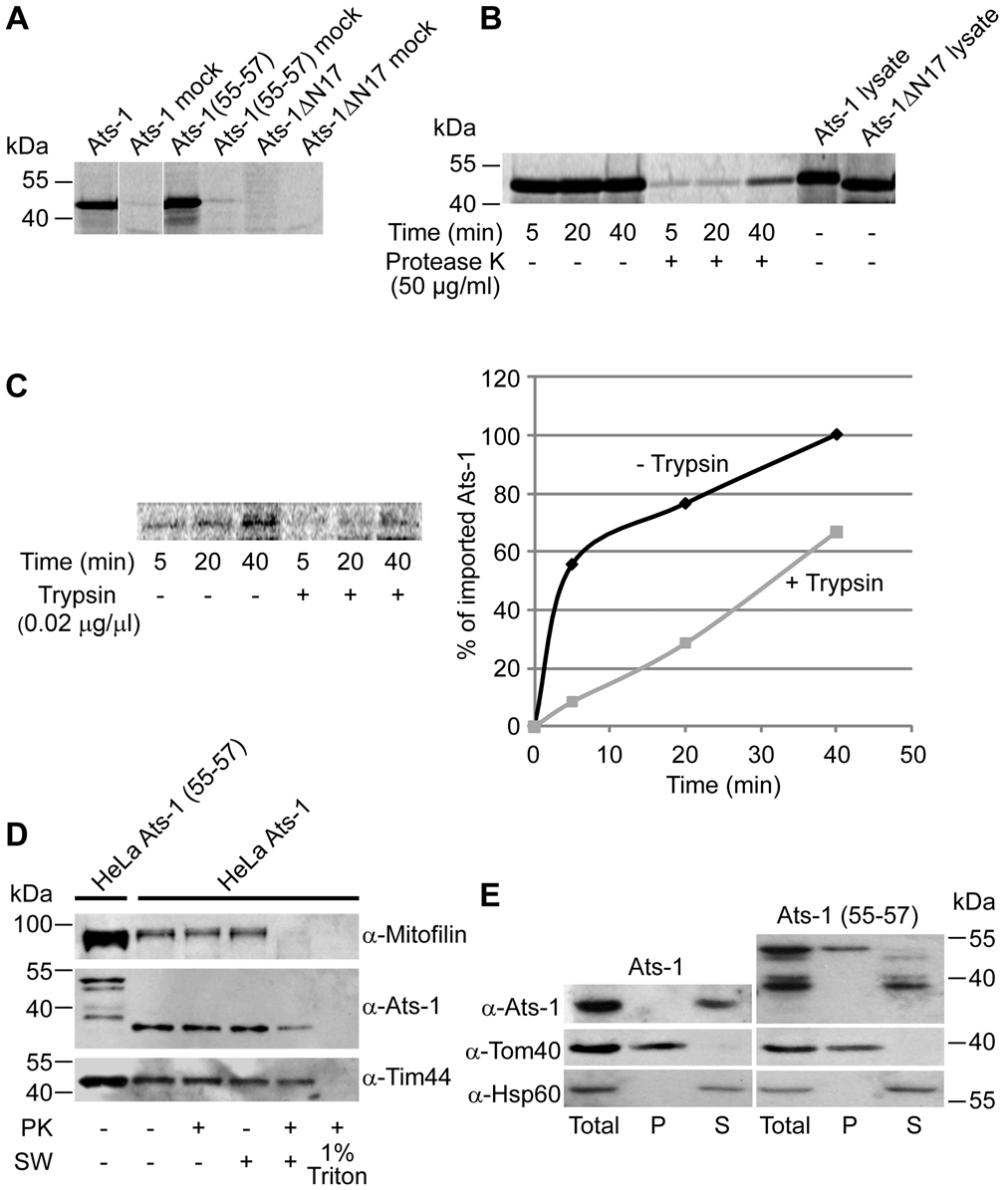
Analysis of the import and sub-mitochondrial localization of Ats-1. **A.** Ats-1, Ats-1 (55–57) and Ats-1 ΔN17 ^35^S-labeled proteins were imported into isolated HeLa mitochondria for 45 min and treated with 50 µg/ml of protease K (PK). Mock samples contain no mitochondria. **B.** ^35^S-labeled Ats-1 was imported into isolated HeLa mitochondria for given time periods, and samples were left untreated (-PK) or treated with 50 µg/ml of protease K (+PK). Two µl of lysate of Ats-1 and Ats-1 ΔN17 was resuspended in Laemmli buffer and analyzed on the gel in addition. **C.** Mitochondria were left untreated (-Trypsin) or treated with 0.02 µg/µl of trypsin (+Trypsin) prior to the import. Import was then performed as in (A) and (B). Signals were quantified using ImageQuant. The import at the longest time point in -Trypsin mitochondria was set as 100%. **D.** HeLa cells were transfected with plasmids containing Ats-1 or Ats-1 (55–57). Mitochondria were isolated after 24 h of overexpression. Mitochondria were incubated in isotonic buffer (- SW), or were swollen in hypotonic buffer (+SW), and then were left untreated (-PK) or treated with 50 µg/ml of protease K (PK). In one sample, mitochondria were solubilized by 1% Triton X-100 (1% Triton), treated with PK and proteins were precipitated using trichloroacetic acid. In addition, 50 µg of mitochondria isolated from cells expressing Ats-1 (55–57) [HeLa Ats-1(55–57)] was resuspended in Laemmli buffer and all samples were analyzed by SDS-PAGE and Western blot, using antibodies against Mitofilin, Tim44 or Ats-1. **E.** Ats-1 in mitochondria behaves as a soluble protein. Ats-1 or a mutant, processing deficient form of the protein Ats-1 (55–57) were expressed in HeLa cells for 24 h. Mitochondria were isolated and subjected to carbonate extraction with 100 mM Na_2_CO_3_, pH 11.5. After centrifugation for 1 h at 100,000 g, pellet (P) and supernatant (S) fraction were separated and, together with total mitochondria, analyzed by SDS-PAGE and Western blot. Membrane was probed with Ats-1, Tom40 and Hsp60 antibodies.

To further analyze mitochondrial targeting and the sub-mitochondrial route of Ats-1, we performed *in vitro* import experiments of ^35^S-labeled Ats-1 and its derivatives Ats-1 (55–57) and Ats-1ΔN17. The ^35^S-labeled proteins were incubated with isolated HeLa mitochondria and mitochondria then were treated with protease K (PK) to remove any non-imported protein. Ats-1 and Ats-1 (55–57) species were detected only in the samples containing mitochondria, and not in the mock samples, where no mitochondria were present, showing that Ats-1 was not simply binding to or exposed to the outside of mitochondria, but was imported into the interior of mitochondria in the PK-inaccessible compartment (Figure 7A). Host cell cytoplasmic modification of Ats-1 was not required for Ats-1 mitochondrial import, as *in vitro* transcribed and translated Ats-1 is imported into mitochondria. Additionally, a PK-resistant protein was not detected when Ats-1ΔN17 was used for import, indicating that the lack of Ats-1ΔN17 targeting to mitochondria is an intrinsic property of this mutant, versus retention of this protein in the cytoplasm of host cells. The amount of PK-undigested Ats-1 increased with time; however, cleavage of the ^35^S-labeled protein was not detectable within 40 min of incubation (Figure 7B).

In general, proteins targeted to mitochondria can take one of several pathways to import into these organelles and sort into the corresponding mitochondrial compartment. The translocase of the outer mitochondrial membrane (TOM) complex is the entry point of practically all mitochondria-targeted proteins. Subsequently, proteins with a cleavable amino-terminal presequence are handed over to the translocase of the inner mitochondrial membrane (TIM) 23 complex and in most cases transported into the mitochondrial matrix, whereas the carrier proteins, with internal targeting signals, are integrated into the inner mitochondrial membrane (IMM) via the TIM22 complex. The outer mitochondrial membrane (OMM) β-barrel proteins are assembled into the OMM with the help of the sorting and assembly machinery complex [34]. The isolated HeLa mitochondria were treated with trypsin prior to import, in order to digest the parts of the TOM import receptors exposed to the cytosol. Significantly less Ats-1 was imported into trypsin-pretreated mitochondria, pointing to the likely involvement of the TOM import receptors in the import of Ats-1 (Figure 7C).

In HeLa cells, both Ats-1 and Ats-1 (55–57) could be found in mitochondria upon over-expression, whereas Ats-1 ΔN17 was found only in the cytosol as in RF/6A cells (Figures 4B, 4C, 4D, and 6F). In all cases, mitochondria retained their usual morphology and membrane potential, as judged by the staining of mitochondria with a membrane-potential sensitive dye MitoTracker (Figure S1). In order to examine the sub-mitochondrial localization of Ats-1 and Ats-1 (55–57), mitochondria were isolated after 24 h of expression in HeLa cells. Ats-1 was cleaved to 35-kDa in HeLa mitochondria as in RF/6A cells (Figure 5C), however, Ats-1 (55–57) showed multiple bands ranging from approximately 48 to 39 kDa, none of them corresponding to the single 35-kDa band that was detected in Ats-1-containing mitochondria (Figure 7D). Thus, in HeLa cells residues 55–57 are critical for proper cleavage of Ats-1 as in RF/6A cells (Figure 6G) or in the case of native Ats-1 (Figure 1B). Next, the 35-kDa Ats-1-containing mitochondria were incubated in an isotonic and a hypotonic buffer, in the presence or in the absence of PK. In addition, mitochondria were solubilized with 1% detergent Triton X-100 and treated with PK. After opening of OMM by swelling of mitochondria in the hypotonic buffer, the IMM protein Mitofilin, which is exposed to the intermembrane space of mitochondria, became sensitive to PK. Tim44, a protein associated with the IMM from the matrix side, remained intact and could be digested only when mitochondria were completely solubilized with Triton X-100. Ats-1 behaved mostly as the matrix protein Tim44, although a portion could be degraded with PK after the swelling of mitochondria, pointing to possible partial IMM localization (Figure 7D).

To further determine the state of Ats-1 sub-mitochondrial localization, mitochondria containing over-expressed Ats-1 and Ats-1 (55–57) were subjected to carbonate extraction with 100 mM Na_2_CO_3_, pH 11.5 to determine whether the protein is membrane integrated or soluble. Integral membrane protein Tom40 was found in the pellet after carbonate extraction, whereas the soluble matrix protein Hsp60 was found in the supernatant. Ats-1 was only detected in the supernatant, pointing to the protein being soluble. For over-expressed Ats-1 (55–57), we could observe two prominent bands, one corresponding to the misprocessed 39-kDa protein and the other at approximately 48 kDa (Figure 7E). The misprocessed protein behaved as a soluble protein, similar to the properly processed Ats-1, whereas the 48-kDa protein was found in the pellet fraction, suggesting that uncleaved Ats-1 (55–57) is stacked in the mitochondrial membrane. We conclude that mature Ats-1 most likely is present as a soluble protein in the matrix of mitochondria, whereas most Ats-1 (55–57) is missorted and misprocessed in the mitochondria.

### Mature Ats-1 inhibits apoptosis in mammalian cells at the mitochondria

**Figure 8:**
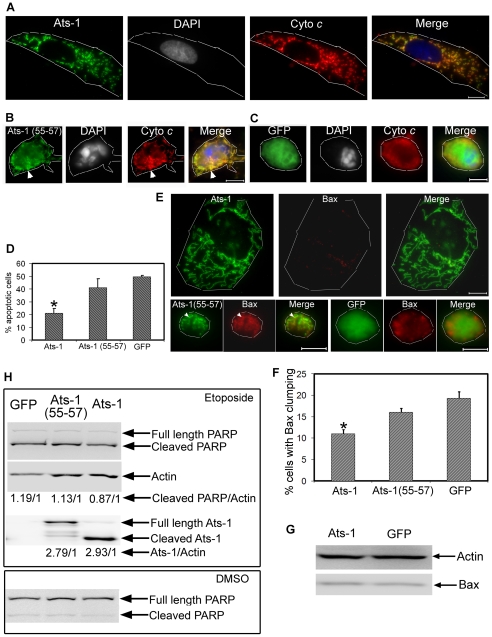
Ats-1 inhibits etoposide-induced apoptosis. **A-C.** Triple fluorescence labeling using rabbit anti-Ats-1 [Ats-1 or Ats-1(55–57); Alexa Fluor 488, green], mouse monoclonal anti-cytochrome *c* (Cyto *c*; Alexa Fluor 555, red), and DAPI (nucleus, gray pseudocolor) in RF/6A cells transfected with pAts-1 (Ats-1) (**A**), pAts-1 (55–57) [Ats-1(55–57)] (**B**), or pGFP (GFP) (**C**), after treatment with etoposide. Scale bar: 10 µm. Note mitochondrial colocalization of Ats-1, a large nucleus, and a spread-out large RF/6A cell in A, and round-up shrunken RF/6A cells with condensed nuclei and presence of diffuse cytochrome *c* released into the cytosol in B and C. Some Ats-1 (55–57) is colocalized with cytochrome *c* retained in the fragmented mitochondria in B (white arrowhead). Contours of host cells are marked with white dashed lines. **D.** Percentage of apoptotic cells in RF/6A cells transfected with pAts-1 (Ats-1), pAts-1 (55–57) [Ats-1(55–57)], or pGFP (GFP) 1 day after etoposide treatment. Data are presented as means ± standard deviations of triplicate samples. *, significantly different compared with GFP-, or Ats-1(55–57)-expressing RF/6A cells by the Tukey's HSD test (*P*<0.01). **E.** Bax staining for RF/6A cells transfected with pAts-1 (Ats-1), pAts-1(55–57) [Ats-1(55–57)], or pGFP (GFP) 1 day after etoposide treatment. Note clumped Bax in the round-up shrunken pAts-1(55–57)- and pGFP-transfected cells, and weak diffuse Bax (almost invisible) in pAts-1-transfected cells, and absence of Bax colocalization with Ats-1 in the mitochondria. In pAts-1(55–57)-transfected cells some Bax is colocalized with Ats-1(55–57) retained in the fragmented mitochondria (white arrowhead). Bar: 10 µm. Contours of host cells are marked with white dashed lines. **F.** Percentage of RF/6A cells transfected with pAts-1(Ats-1), pAts-1(55–57) [Ats-1(55–57)], or pGFP (GFP), showing obvious Bax redistribution (clumping) 1 day after etoposide treatment. Data are presented as means ± standard deviations of triplicate samples. *, significantly different compared with GFP-, or Ats-1(55–57)-expressing RF/6A cells by the Tukey HSD test (*P*<0.01). **G.** Bax amount in RF/6A cells transfected with pAts-1(Ats-1), or pGFP (GFP) 1 day after etoposide treatment. Actin is used to normalize the protein loading amount. **H.** Western blot analysis for cleavage of PARP in RF/6A cell transfected with pAts-1 (Ats-1), pAts-1(55–57) [Ats-1(55–57)], or pGFP (GFP) after treatment with etoposide or DMSO for 12 h. Samples were subjected to probing with anti-PARP, anti-actin, or anti-Ats-1 by Western blot analysis. Full-length and cleaved PARP, actin, full-length and cleaved Ats-1 are indicated with arrows. Relative density ratios of cleaved PARP/actin bands, or total Ats-1 (full length, cleaved and degraded Ats-1)/actin bands are shown below each lane in etoposide-treated group.

*A. phagocytophilum* infection inhibited mitochondria-mediated apoptosis in human neutrophils [12]. Given that Ats-1 was routed to the mitochondrial matrix in *A. phagocytophilum*-infected cells, we wondered whether mature Ats-1 is involved in apoptosis inhibition. Etoposide, a topoisomerase II inhibitor, causes DNA damage leading to the activation of caspase 2 and subsequent induction of Bax translocation to mitochondria and cytochrome *c* release [35]. Therefore, we treated pGFP-, pAts-1-, or pAts-1 (55–57)-transfected RF/6A cells with etoposide. Ats-1 and Ats-1 (55–57) localized in mitochondria as shown in Figures 4 and 6, but GFP showed diffuse distribution. Most of the Ats-1-expressing RF/6A cells were spread-out flat with a homogeneously oval shape of nucleus, and elongated mitochondria retaining both Ats-1 and cytochome *c* at day 1 after treatment with etoposide (Figures 8A and D). However, ∼50% of GFP-expressing RF/6A cells and ∼40% of Ats-1 (55–57)-expressing RF/6A cells were apoptotic as judged by rounded shrunken cell morphology, condensed and fragmented nuclei, and release of cytochrome *c* into the cytosol (diffuse staining, instead of distinct mitochondrial staining) (Figures 8B, 8C, and 8D). In pAts-1 (55–57)-transfected cells, both Ats-1 (55–57) and cytochrome *c* appeared to be retained in some fragmented mitochondria (Figure 8B).

The pro-apoptotic Bcl-2 family protein Bax is found mainly in the cytoplasm or loosely attached to OMM as inactive monomers in non-apoptotic cells [36]. Following any one of various cytotoxic signals, Bax is activated and undergoes a series of conformational changes in the N- and C-termini, leading to Bax translocation to the mitochondria, oligomerization, and integration into the mitochondrial membranes [37],[38]. These events have all been implicated in the process of cytochrome *c* release, although the precise biochemical sequence of events for Bax redistribution to mitochondria and cytochrome *c* release is still unclear [39]. One day after treatment with etoposide, Bax redistribution to mitochondria containing Ats-1 (55–57) and Bax clumping in GFP-expressing apoptotic RF/6A cells were observed (Figures 8E and 8F). As it was difficult to recognize clumped Bax in many apoptotic cells, we scored only the cells that we could recognize with confidence. Bax was so diffuse in the thinly spread cytoplasm of Ats-1-transfected cells that it was barely visible even after etoposide treatment (Figure 8E). It is important to note that the total cellular Bax amount was not obviously different between Ats-1- and GFP-transfected RF/6A cells after etoposide treatment (Figure 8G). From these studies, we concluded that mature Ats-1 inhibits Bax redistribution.

The release of cytochrome *c* from mitochondria is an important step in mitochondria-mediated apoptosis, leading to activation of caspase 9 and then caspase 3 [40]. Poly (ADP-ribose) polymerase (PARP) is involved in DNA repair in response to environmental stress [41]. During apoptosis, full length PARP (116 kDa) is cleaved into an 89-kDa fragment by caspase 3 [42],[43]. The relative ratio of cleaved PARP to actin was lower in Ats-1-transfected RF/6A cells than in GFP-, or Ats-1 (55–57)-transfected RF/6A cells 12 h after etoposide treatment (Figure 8H). However, the expression levels of Ats-1 and Ats-1 (55–57) in RF/6A cells were similar (Figure 8H). This result is in agreement with immunofluorescence labeling of cytochrome *c*, DAPI, and Bax (Figures 8A-F), indicating that mature Ats-1 in the mitochondria inhibits etoposide-induced apoptosis. Without etoposide treatment, there is only minor cleavage of PARP after transfection with these plasmids (Figure 8H).

### Ats-1 inhibits Bax-induced apoptosis in yeast cells

**Figure 9:**
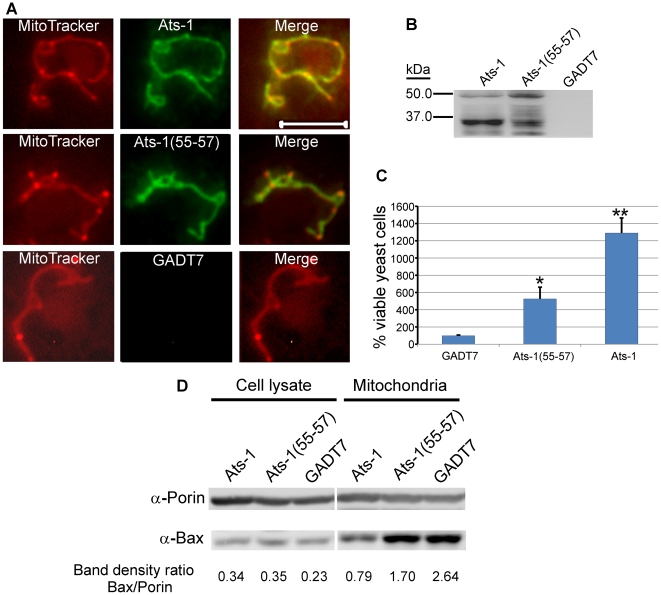
Ats-1 inhibits Bax-induced apoptosis in yeast. **A.** Mitochondrial colocalization of Ats-1 and Ats-1(55–57) in *S. cerevisiae*. pYAts-1 (Ats-1), pYAts-1(55–57) [Ats-1(55–57)], or pGADT7 AD(GADT7)–transformed YPH499 yeast cells were loaded with MitoTracker Red, and subjected to immunostaining using rabbit anti-Ats-1, and Alexa Fluor 488-conjugated goat anti-rabbit IgG. Scale bar: 5 µm. **B.** Expression of Ats-1, or Ats-1(55–57) in *S. cerevisiae*. The cell lysates of YPH499 cells transformed with pYAts-1 (Ats-1), pYAts-1(55–57) [Ats-1(55–57)], or pGADT7 AD(GADT7) were subjected to Western blot analysis using anti-Ats-1. Note 35-kDa mature Ats-1 from wild type Ats-1 and abnormal cleavage of Ats-1(55–57). **C.** Ats-1 partially rescues *S. cerevisiae* from Bax-induced growth arrest. Yeast YPH499 cells were co-transformed with pBax, and pGADT7 AD (GADT7), pYAts-1(55–57) [Ats-1(55–57)], or pYAts-1(Ats-1). Recombinant yeast cells were cultured in SG medium containing galactose to induce Bax expression. The number of viable yeast cells was determined by plate count technique. The numbers of viable cells at day 5 after Bax induction were compared to the viable cells at day 0. Data are presented as means and standard deviations of three independent experiments. * and **, Significantly different among each other by Tukey's HSD test (*P*<0.05). **D.** Translocation of Bax to mitochondria in *S. cerevisiae.* After induction to express Bax for 12 h, the total cell lysates and mitochondria isolated from YPH499 cells co-transformed with pBax, and pGADT7 AD (GADT7), pYAts-1(55–57) [Ats-1(55–57)], or pYAts-1(Ats-1), were subjected to Western blot analysis using anti-Bax, and anti- *S. cerevisiae* Porin. Relative density ratios of Bax/Porin bands are shown below each lane.

Although yeast lacks Bcl-2 members, Apaf-1, and p53, the cell death-regulating activity of Bcl-2 members is conserved in yeast and it is well known that heterologous expression of human Bax induces growth arrest and cell death in yeast cells [44]. Human Bax translocates to yeast mitochondria and induces apoptotic changes, which can be prevented by human Bcl-X_L_ co-expression [44],[45]. To further analyze the anti-apoptotic mechanism of Ats-1 at mitochondria, we tested whether Ats-1 and Ats-1 (55–57) could target mitochondria in *Saccharomyces cerevisiae*, and reduce sensitivity to the apoptosis induced by human Bax. Both Ats-1 and Ats-1 (55–57) localized to the yeast mitochondria (Figure 9A), indicating the robust Ats-1 mitochondria localization signal. Also in yeast, over-expressed Ats-1 was more effectively cleaved to the 35 kDa size as observed in mammalian mitochondria, suggesting it was imported to the matrix. In contrast, Ats-1 (55–57) cleavage was less pronounced and aberrant, suggesting a limited accessibility to or recognition by the matrix protease (Figure 9B). Ats-1, and, to a significantly lesser extent, Ats-1 (55–57), could partially rescue *S. cerevisiae* from Bax-induced apoptosis (growth arrest and death) (Figure 9C). While the total Bax expression level did not change, Bax translocation to yeast mitochondria was reproducibly reduced by Ats-1 as compared to Ats-1 (55–57) or control plasmid (Figure 9D). *S. cerevisiae* porin was used as loading control for the amount of mitochondria in Western blot analysis. The transferred membrane was also stained with ponceau S to compare total protein loading amount and profile among samples (Figure S2). Taken together, these results suggest mature Ats-1 in the matrix renders mitochondria resistant to Bax docking, resulting in yeast rescue.

## Discussion

Our result showed that native Ats-1 is secreted and localizes to the mitochondria in infected human neutrophils and HL-60. It is important to note that Ats-1 is abundant, similar to AnkA [21], allowing us to detect the native protein in the natural host (human neutrophils). The abundance of Ats-1 and AnkA suggests that these *A. phagocytophilum* T4S substrates are not toxic to the host cells, but rather harness or promote intracellular survival and growth of bacteria at the expense of substantial bacterial energy. This is distinct from many substrates of T4S of *Legionella*, *Coxiella*, *Bartonella*, or *Brucella* in which secretion was detected by using transformed bacteria [46],[47],[48],[49]. Notably, when fused with GFP tag, Ats-1 did not localize to the mitochondria, underscoring the importance of verifying the ectopic expression results with the native protein in the natural host. Timing of mitochondrial translocation of Ats-1 was mid-log phase of the *A. phagocytophilum* intracellular growth, approximately coinciding with the up-regulation of VirB9 and VirB6, components of the T4S apparatus of *A. phagocytophilum* [20]. Similar to *A. phagocytophilum* T4S substrate AnkA [21], the C-terminus of Ats-1 contains basic amino acids. Further studies are needed to characterize the consensus translocation signal for *A. phagocytophilum* T4S substrates. Unlike AnkA, no phosphorylation or other host signaling motifs have been detected in Ats-1 thus far, except the mitochondrial localization signal.

Ats-1 is the first example of a bacterial protein that traverses five membranes to localize in the mitochondrial matrix. Ectopically expressed Ats-1 targets mitochondria in various mammalian cells and yeast like native *A. phagocytophilum* Ats-1. Furthermore, *in vitro* translated Ats-1 was translocated to isolated mitochondria in a cell-free system, indicating that Ats-1 alone is sufficient for sub-mitochondrial targeting. Most mitochondrial proteins are encoded in the nucleus and synthesized by ribosomes in the cytoplasm as precursor proteins containing cleavable N-terminal presequence essential for mitochondrial targeting [33]. Our results showed that the 17 N-terminal amino acids of Ats-1, as part of the presequence, are critical for mitochondrial targeting. Deletion of this sequence not only prevented mitochondrial targeting, but subsequently prevented presequence cleavage. The presequence is cleaved from preproteins by the matrix-located mitochondrial processing peptidase after import into the mitochondrial matrix. Presequences vary in length and share little sequence similarity. However, a high content of positively charged residues and a capacity to form an amphipathic α-helix are common characteristics [33]. In addition, cleavage sites commonly contain a basic amino acid residue, usually arginine, at the -2 position [50]. Our result showed that the cleavage site of Ats-1 is between residues 45–60, and further, that residues 55–57 are crucial in facilitating proper cleavage. Since only one arginine at position 58 is present in this segment between residues 45–60 of Ats-1, it suggests that cleavage may occur between residues 59 and 60.

Ats-1 is likely to follow the classical mitochondrial import route, involving the TOM complex in the OMM. Indeed, we could show that Ats-1 import likely depends on the TOM import receptors. Interestingly, although mostly processed Ats-1 was detected in mitochondria after the over-expression of the protein for 24 h in HeLa, RF/6A, and yeast cells, we could not see any processing of the presequence of Ats-1 after *in vitro* import. One possible reason for this is that the processing of Ats-1 takes longer than the 40 min time-period that we could observe in *in vitro* import experiments. In this aspect, Ats-1 is different from endogenous mitochondrial presequence-containing proteins, where the processing of the presequence takes place within minutes upon import [51]. Related to mitochondrial transport, it was reported that the depletion of either Tom40 or Tom22 reduces *Chlamydia caviae* infection in HeLa cells by unknown mechanisms [52]. Effectors targeting host cell mitochondria have been described previously for pathogenic bacteria utilizing a type III secretion system [53],[54],[55]. However, the mitochondrial localization routes are not known. In addition, their biological functions are opposite to Ats-1: enteropathogenic *Echerichia coli* EspF is required to initiate apoptosis [53]. Moreover, several effectors of *Pseudomonas syringae* are deleterious when expressed in yeasts [54]. The biological function of SopA from *Salmonella enterica* colocalized with mitochondria is unknown [55].

Recently, two T4S substrates were shown to target mitochondria: when ectopically expressed in HeLa cells, *Coxiella burnetii* AnkJ-mCherry colocalized to the mitochondria [47], although where and how AnkJ is localized in the mitochondria and whether AnkJ localizes in mitochondria of infected cells is unknown. Ectopically expressed His- LegS2 and Myc-tagged LegS2 secreted by *L. pneumophila* surround the mitochondria from the outside and form patches on the mitochondrial outer membrane [56]. How LegS2 is localized to mitochondria is unknown. Furthermore, what the exact contributions of AnkJ and LegS2 to the pathogenesis are not known. Our results with mitochondrial fractionation and carbonate extraction point to Ats-1 being a soluble or loosely membrane-associated matrix protein. This is not surprising, considering that the protein possesses a cleavable amino-terminal presequence, which is a hallmark of most mitochondrial matrix proteins. To our knowledge, Ats-1 is the first bacterial T4S substrate with a robust targeting presequence to access the mitochondrial matrix, which may serve as a tool to dissect exogenous protein import in the mitochondria.

Although not localized to mitochondria, several bacterial proteins can inhibit apoptosis of eukaryotic cells, at upstream of mitochondria by various mechanisms [57]. The *C. trachomatis* protease, CPAF secreted into the host cell cytosol by an unknown mechanism, can degrade the pro-apoptotic BH3-only proteins [58]. However, it remains to be determined whether CPAF alone in the absence of chlamydial infection can degrade the BH3-only proteins inside cells and prevent host cells from undergoing apoptosis. *C. trachomatis* recruits BAD to its vacuole by unknown mechanism, which correlates with host-cell survival [59], and infection-induced upregulation of IAPs and Mcl-1 is required to keep the cells in an apoptosis resistant state [60],[61]. Ectopically expressed BepA of *B. henselae* localizes to the plasma membrane and inhibits vascular endothelial cell apoptosis with a concomitant increase in cellular cyclic AMP [17]. Macrophages infected with an *L. pneumophila sdhA* mutant undergo apoptosis [16]. Which subcellular compartment SdhA is localized, and how this protein acts to block cell death activity remain to be determined. Mouse macrophages infected with an *L. pneumophila* SidF mutant also undergo apoptosis, and HeLa cells ectopically expressing *L. pneumophila* SidF are resistant to apoptosis induced by staurosporine. Ectopically expressed SidF localizes to the ER, and SidF interacts with pro-death members of the Bcl2 protein family, BNIP3 and Bcl-rambo as shown by various methods including U937 cells infected with a SidF overproducing strain, although this interaction was not detected with the wild-type strain [18]. PorB, outer membrane protein of *Neisseria meningitides* is the only bacterial protein known to target mitochondria and inhibit apoptosis [62]. Upon incubation of mammalian cells with isolated PorB, it targets and binds mitochondrial outer membrane VDAC (voltage-dependent anion channel), and inhibits host cell apoptosis [62]. Ectopically expressed Ats-1 directly translocates to the mitochondrial matrix and inhibits Bax translocation and cytochrome *c* release from the mitochondria following treatment with etoposide. Bax-induced, mitochondria-mediated yeast apoptosis was also abrogated when Ats-1 was localized in mitochondria. These are not isolated events with ectopic expression, but rather coincide with cellular events observed in human neutrophils infected with *A. phagocytophilum* [12],[13]. In view of the likely matrix/inner membrane localization of Ats-1, it is possible that Ats-1 stabilizes the mitochondrial membrane potential or even the inner-membrane cristae structure (the rearrangement of which was shown to be required for apoptosis [63]). Since soluble mature Ats-1 is more effective in apoptosis inhibition than insoluble and mis-cleaved Ats-1 (55–57), Ats-1 sub-mitochondrial routing and/or processing seem to be critical for proper function. Unlike vMIA of cytomegalovirus, which is a mitochondrial OM-localized inhibitor of apoptosis that causes mitochondrial fragmentation [64], over-expressed Ats-1 did not have obvious adverse effects on mitochondria in RF/6A, HeLa, HL-60, or yeast cells. It is possible that Ats-1 has additional roles in facilitating *A. phagocytophilum* infection.

Mature Ats-1 in mitochondria inhibited Bax redistribution to these organelles. Since Bax is a cytosolic protein and Bax translocation to mitochondria is often considered to be regulated by the Bcl-2 family proteins and, therefore, is separated spatially from the mitochondrial matrix protein, Ats-1, we want to specifically address this issue. First, in the absence of Bcl-2 family proteins in yeast, Ats-1 inhibited Bax docking to the mitochondria and apoptosis, indicating that other Bcl-2 family members are not important in this process. Second, alterations in mitochondrial physiological conditions regulate Bax docking to mitochondria. For example, neither an uncoupler of oxidative phosphorylation, FCCP nor the F(1)-F(0) ATPase inhibitor, oligomycin alone alters Bax location in mammalian cells. However, the combination of the mitochondrial uncoupler FCCP and oligomycin, or compounds that collapse mitochondrial membrane potential such as antimycin and rotenone, trigger Bax docking to mitochondria [65]. Translocation/activation of Bax occurs not only upstream of cytochrome *c* release, but also downstream of cytochrome *c* release and caspase activation [39],[66]. Bax insertion and oligomerization are also influenced by the lipids in the mitochondrial outer membrane [67]. Studies primarily showed Bax association with mitochondrial fission sites with the inner membrane protein mitofilin and dynamin-related protein 1 [65]. Bcl2 and Bax have been found at the mitochondrial contact sites [68],[69] where the outer and inner mitochondrial membranes are in close contact and are virtually indistinguishable. Thus, it is possible that mature Ats-1 in the matrix may loosely associate with inner membrane and stabilize membrane potential, influence mitochondrial energizing, or mitochondrial membrane conformation, making them more resistant to Bax docking. Since Ats-1 is the first bacterial T4S protein targeted to the mitochondrial matrix, detailed mechanisms of apoptosis inhibition by Ats-1 at mitochondria deserve further study.

In conclusion, our data present the first example of a bacterial protein that traverses five membranes via a characterized route and processing to inhibit apoptosis at mitochondria. In the future, vaccines or drugs designed to target the anti-apoptotic mechanism of *A. phagocytophilum* Ats-1 may prevent this organism from proliferating in its host cells, leading to the control of human granulocytic anaplasmosis. The absence of sequence similarity or mode of action to any other known cell death suppressors suggests that Ats-1 defines a previously undescribed class of anti-apoptotic protein.

## Materials and Methods

### GenBank accession number

VirD4: YP_505894; APH0859: YP_505436; VirB11: YP_505895; AM410: YP_153722; *ats-1*: FJ210653.

### Anaplasma phagocytophilum, isolation and cell culture

*A. phagocytophilum* HZ strain was propagated in HL-60 cells (American Type Culture Collection (ATCC), Manassas, VA) in complete RPMI 1640 medium (Invitrogen, Carlsbad, CA) supplemented with 10% fetal bovine serum (US Biotechnologies, Parkerford, PA) and 2 mM l-glutamine (Invitrogen). RF/6A monkey endothelial cells (ATCC) were cultured in advanced MEM (Invitrogen), supplemented with 10% fetal bovine serum and 2 mM l-glutamine. Cultures were incubated at 37°C under 5% CO_2_ in a humidified atmosphere. Human neutrophils were isolated by Dextran sedimentation followed by Hypaque gradient centrifugation, and subjected to infection with *A. phagocytophilum*, as described previously [20]. Host cell-free *A. phagocytophilum* was prepared by sonication or nitrogen cavitation, as described elsewhere [20]. For cellular fractionation, 1.5×10^8^ *A. phagocytophilum*–infected HL-60 cells were resuspended in 25 ml RPMI 1640 medium and subjected to nitrogen cavitation. The cellular fraction containing *A. phagocytophilum* and mitochondria was pelleted by centrifugation at 10,000×*g* for 10 min at 4°C. *A. phagocytophilum* was further purified from the pellet by Percoll density-gradient centrifugation, as described elsewhere [70]. HeLa cells were grown in RPMI 1640 (Gibco) supplemented with 10% fetal calf serum (Biochrom) and penicillin/streptomycin.

### Bacterial two-hybrid system

Genomic DNA was isolated from Percoll density gradient–purified *A. phagocytophilum*. The DNA fragment encoding residues 2-740 of VirD4 was amplified by PCR using the forward primer 5′-AGTGCGGCCGCACATAGTTCCAATCATATACGAAA-3′, (Not I site is underlined) and the reverse primer 5′-TTACTCGAGCTACTTTAGTCTTCCGTTACT-3′ (Xho I site is underlined) from genomic DNA. The PCR product was digested with Not I and Xho I and ligated into the pBT plasmid (BacterioMatch II two-hybrid system; Stratagene, La Jolla, CA). Ligated products were transformed into XL1-Blue (MRF' Kan strain; Stratagene), resulting in recombinant bait plasmid, pBT-VirD4.

To construct the random genomic DNA library of *A. phagocytophilum*, the pTRG prey plasmid from the BacterioMatch II two-hybrid system was first modified by introducing two BstXI sites into the multiple cloning sites. To insert the first BstXI site, PCR amplification was performed using pTRG as a template and the following pair of primers: pTRG BstXI Forward I, 5′-CGAGGAT*CC* *AGTGTGGTGG* CGGCCGCAAGAATTCAGTCT-3′ (BamH I and Not I sites are underlined, BstXI site is italicized) and pTRG BstXI Reverse I, 5′-CGCGGATCCGGCCGCCTCTGGTTTCTCTT-3′ (BamH I site is underlined). The PCR product was digested with BamH I and self-ligated, resulting in the insertion of the first BstXI site between the BamH I and Not I sites in pTRG vector. For the insertion of the second BstXI site, PCR amplification was performed using the partially modified pTRG as template and the second pair of primers: pTRG BstXI Forward II: 5′-CGACTCGAG *CCAGCACAGTG*TAATTAATTAATTAATGAACTAGTGAGATC-3′ (Xho I site is underlined and BstXI site is italicized) and pTRG BstXI Reverse II 5′-CGCCTCGAGCGCCAGCTCAGACTGAATTC-3′ (Xho I site is underlined). The PCR product was digested with Xho I and self-ligated, resulting in the insertion of the second BstXI site downstream of the Xho I site.

*A. phagocytophilum* genomic DNA (10 µg) was fragmented by nebulization (Invitrogen) according to the manufacturer's instruction. Fragmented DNA (0.5 to 3.0 kb) was ligated with a BstXI adapter (Invitrogen) after its termini were repaired and phosphorylated. BstXI-adapted genomic DNA fragments were ligated into BstXI-digested modified pTRG vector and transformed into XL1-Blue MRF' Kan cells by electroporation, according to the manufacturer's instruction (MicroPulser Electroporator, Bio-Rad, Hercules, CA). After incubation at 30°C for 24 h, transformants were harvested and recombinant plasmids were purified using a QIAprep Maxi kit (Qiagen).

### Screening of the random genomic DNA library for VirD4-interacting protein

A mixture of 50 ng pBT-VirD4 vector and 50 ng pTRG library DNA was transformed into BacterioMatch II electrocompetent reporter cells (Stratagene) via electroporation. Transformants were screened according to the manufacturer's instruction. Positive interactions were identified by growth on selective screening medium consisting of minimal medium plus 5 mM 3-amino-1,2,4-triazole (3-AT), 25 ng/ml chloramphenicol and 12.5 ng/ml tetracycline for 40 h (24 h at 37°C, and then 16 h at room temperature) and validated by growth on dual selective screening medium consisting of minimal medium plus 5 mM 3-AT, 12.5 ng/ml streptomycin, 25 ng/ml chloramphenicol and 12.5 ng/ml tetracycline. All media were prepared according to the manufacturer's instruction (BacterioMatch II two-hybrid system; Stratagene). False positive controls for bait and prey were performed using pBT-VirD4 and empty prey vector, or empty bait vector and pTRG-prey.

### Expression of recombinant APH0859, antibody production, and affinity purification

A 765-bp DNA fragment encoding APH0859 was amplified by PCR using *A. phagocytophilum* genomic DNA as template, and cloned into pET33b (+) protein expression vector (Novagen, Madison, WI). Primer set (APH0859 Expression Forward, and APH0859 Expression Reverse) used in this PCR is listed in Supplementary data (Table S1). The recombinant plasmid was transformed into *E. coli* BL21(DE3) (Novagen) and cultures were grown in Luria–Bertani (LB) medium. The expression of recombinant APH0859 (rAPH0859) was induced at 25°C for 5 h by addition of isopropyl-beta-D-thiogalactopyranoside (1 mM final). rAPH0859 was affinity-purified using a HisBind Quick Column under non-denaturing conditions as instructed by the manufacturer (Novagen). The purified protein was subjected to SDS-PAGE analysis and the rAPH0859 band was excised and used to prepare antiserum in rabbits (Prosci Incorporated, Poway, CA). For affinity purification of the anti-rAPH0859 serum, 3 ml anti-rAPH0859 serum was incubated with 1 ml of rAPH0859-conjugated Affi-Gel 10 (Bio-Rad). After washing with TBS (150 mM NaCl, 50 mM Tris-HCl, pH 7.4), the antibody was eluted using 0.1 M glycine-HCl, pH 2.5. Eluted antibody was neutralized by addition of 0.1 volume of 1.0 M Tris-HCl, pH 8.8.

### Protein identification by mass spectrometry

*A. phagocytophilum*–infected HL-60 cells (1.0×10^8^ cells) were pelleted by centrifugation, washed twice with cold PBS buffer (137 mM NaCl, 2.7 mM KCl, 10 mM Na_2_HPO_4_, and 2 mM KH_2_PO_4,_ pH 7.4), and lysed in 2.0 ml cold lysis buffer (150 mM NaCl, 1 mM EDTA, 20 mM Tris-HCl, pH 7.5) containing 1% (v/v) Triton X-100 and 2% (v/v) protease inhibitor cocktail set III (EMD Chemicals, San Diego, CA). The lysate was pre-cleared with 100 µl protein A-agarose (Santa Cruz Biotechnology, Santa Cruz, CA), and incubated with 50 µg affinity-purified rabbit anti-rAPH0859 antibody or rabbit pre-immune IgG for 2 h at 4°C. The immune complex was precipitated by addition of 300 µl protein A-agarose and incubated overnight. The protein A-agarose was washed four times with PBS, and bound protein was eluted by the addition of 100 µl SDS-PAGE loading buffer (4% SDS, 135 mM Tris-HCl, pH 6.8, 10% (v/v) glycerol, and 10% (v/v) β-mercaptoethanol) and boiled for 5 min. Proteins were separated by SDS-PAGE and the 48-kDa band, which was specifically immunoprecipitated by anti-rAPH0859 antibody, was excised, digested with chymotrypsin, and subjected to protein identification using LC-MS-MS (Mass Spectrometry & Proteomics Facility at Ohio State University) after fixation and staining with GelCode Blue (Pierce).

### Time-course infection by *A. phagocytophilum*

Infection of HL-60 cells or human neutrophils with host cell-free *A*. *phagocytophilum* was performed as described previously with minor modifications [20]. Briefly, human neutrophils or HL-60 cells (1.5×10^7^ cells) were incubated with *A. phagocytophilum* purified from 2×10^7^ infected HL-60 cells in 15 ml complete RPMI 1460 medium (approximate bacteria to host cell ratio of 100∶1). After 2 h incubation at 37°C, infected cells were washed twice with PBS to remove free bacteria, and then incubated at 37°C for the designated time period. *A. phagocytophilum-*infected neutrophils or HL-60 cells were fixed with paraformaldehyde and were subjected to immunofluorescence labeling.

### Construction of recombinant mammalian expression plasmids for transfection

All primers used for construction of mammalian expression plasmids are listed in Table S1. To obtain full-length *ats*-1, the primers Ats-1 Forward and Ats-1 Reverse were used. Ats-1 ΔN17 was prepared using the primers Ats-1 delta N17 Forward and Ats-1 Reverse. To insert the sequence encoding an HA tag within *ats-1*, the DNA fragment encoding the C-terminal Ats-1 segment (HA C1) was amplified using the primers HA Forward C1 and Ats-1 Reverse. Using the HA C1 fragment as template and the primers HA Forward C2 and Ats-1 Reverse, HA C2 was amplified. Using the HA C2 fragment as template and the primers HA Forward C3 and Ats-1 Reverse, HA C3 was amplified. The DNA fragment encoding the N-terminal Ats-1 segment (HA N) was amplified using the primers Ats-1 Forward and HA Reverse N. Finally, based on the complimentary DNA sequence at the 3′-terminus of HA N and the 5′-terminus of HA C3, the two DNA fragments were linked by PCR, resulting the insertion of HA tag within Ats-1. To prepare Ats-1 mutants containing substitutions at residues 46–60, the primers Ats-1 forward and AAA (or AGA) Reverse were used to generate fragments encoding the N-terminus of Ats-1, and the primers AAA (or AGA) Forward and Ats-1 Reverse were used to generate fragments encoding the C-terminus of Ats-1. Based on the complimentary DNA sequence these two DNA fragments were linked by PCR amplification, resulting in the amino acid substitutions in Ats-1. All of PCR products were cloned into pEGFP-N1 vector which was pre-digested with SalI and NotI to remove the GFP-encoding sequence (Clontech), resulting in the following recombinant plasmids. pAts-1, encoding full-length Ats-1; pAts-1 (30HA), encoding Ats-1 with the HA insertion between residues 30 and 31; pAts-1 (45HA), encoding Ats-1 with the HA insertion between residues 45 and 46; pAts-1 (60HA), encoding Ats-1 with the HA insertion between residues 60 and 61; pAts-1 (72HA), encoding Ats-1 with the HA insertion between residues 72 and 73; pAts-1 (NGL46-48AAA), encoding Ats-1 with AAA substitutions at residues 46–48; pAts-1 (MGK49-51AAA), encoding Ats-1 with AAA substitutions at residues 49–51; pAts-1 (GKP52-54AAA), encoding Ats-1 with AAA substitutions at residues 52–54; pAts-1 (FYH55-57AAA), encoding Ats-1 with AAA substitutions at residues 55–57; pAts-1 (RAS58-60AGA), encoding Ats-1 with AGA substitutions at residues 58–60; pAts-1 ΔN17, encoding Ats-1 with the deleted mitochondrial targeting sequence.

### Mitochondrial import and sub-mitochondrial localization of Ats-1

Using pAts-1, pAts-1 (55–57), and pAts-1ΔN17 as template, and primers (Ats-1 Free N Forward, Ats-1 Free N delta 17 Forward, and Ats-1 Free N Reverse) listed in the Table S1, *ats-1*, *ats-1* (55–57) and *ats-1* ΔN17 were cloned into the pET41(+) vector (Novagen). *In vitro* transcription/translation from the T7 promoter was performed using TnT Coupled Reticulocyte Lysate System (Promega) in the presence of ^35^S-methionine/cysteine (Perkin-Elmer) according to the manufacturer's instruction. The final protein products contain additional 8 histidines at the carboxy-terminus derived from vector. Mitochondria isolation and *in vitro* import were performed as described previously [51]. Import samples were subsequently treated with 50 µg/ml of protease K, which was inhibited by the addition of 2 mM phenylmethylsulphonyl fluoride (PMSF).

For trypsin-pretreatment of mitochondria, isolated mitochondria were treated with 0.02 µg/µl of trypsin for 10 min on ice. Trypsin was inhibited by the addition of 30-fold excess of soybean trypsin inhibitor (STI). In the control (- trypsin) samples, STI was added before the addition of trypsin. Mitochondria were reisolated, washed once with sucrose buffer (250 mM sucrose, 10 mM Tris, pH 7.6, 1 mM EDTA, pH 8.0) with addition of 1 mg/ml STI, and used for *in vitro* import. ^35^S-labeled proteins were detected using autoradiography and signals were quantified using the ImageQuant program.

For swelling of mitochondria, mitochondria were incubated in isotonic sucrose buffer, or hypotonic ET buffer (10 mM Tris, pH 7.6, 1 mM EDTA, pH 8.0) for 30 min on ice, reisolated, resuspended in 100 µl of sucrose buffer and treated with 50 µg/ml of PK where indicated. For complete solubilization of mitochondria, 1% Triton X-100 in detergent buffer (20mM Tris–HCl, 0.1 mM EDTA, 1 mM PMSF, 50 mM NaCl, 10% (v/v) glycerol, pH 7.4) was used. Samples were then treated with 50 µg/ml of PK and proteins were precipitated using trichloroacetic acid.

To determine the sub-mitochondrial location of Ats-1, mitochondria were isolated from Ats-1, or Ats-1 (55–57)-transfected HeLa cells, and subjected to carbonate extraction with 100 mM Na_2_CO_3_, pH 11.5. After centrifugation for 1 h at 100,000×g, pellet and supernatant fractions were separated and, together with total mitochondria, analyzed by SDS-PAGE and Western blot. The membrane was probed with Ats-1, Tom40 and Hsp60 antibodies.

### Immunofluorescence microscopy

For immunofluorescence labeling in HL-60 and RF/6A cells, cells were fixed with 2% paraformaldehyde in PBS at RT for 25 min and then washed three times with PBS before labeling. Permeabilization and blocking were performed using PGS solution (PBS containing 0.4% BSA, 0.2% gelatin and 0.3% saponin) at RT for 1 h. For the determination of Ats-1 translocation in *A. phagocytophilum*-infected HL-60 cells, double immunofluorescence labeling was performed using rabbit anti-Ats-1 and monoclonal 5C11 (anti-P44) antibodies [28], with Alexa Fluor 555-conjugated goat anti-rabbit IgG and Alexa Fluor 488-conjugated goat anti-mouse IgG (Molecular Probes) as secondary antibodies. To investigate the targeting of translocated Ats-1 to mitochondria in *A. phagocytophilum*-infected HL-60 cells or human neutrophils, triple immunofluorescence labeling was performed using horse anti-*A. phagocytophilum* serum, affinity-purified rabbit anti-Ats-1 antibody, and mouse monoclonal anti–Mn-Sod antibody (clone MnS-1, Alexis, San Diego, CA), with Affipure Cy3-conjugated goat anti–horse IgG (Jackson ImmunoResearch Laboratories), Alexa Fluor 488–conjugated goat anti-rabbit IgG and Alexa Fluor 350-conjugated goat anti-mouse IgG (Molecular Probes) as secondary antibodies. To stain Mn-Sod, Bax, or cytochrome *c* in pAts-1-, pAts-1 (55–57), or pGFP-transfected RF/6A cells, cells were labeled with rabbit anti-Ats-1 and mouse monoclonal anti–Mn-Sod, mouse monoclonal anti-Bax (Clone 3, BD Transduction Laboratory), or anti-cytochrome *c* (clone 2G8, Santa Cruz biotechnology), with Alexa Fluor 488-conjugated goat anti-rabbit IgG and Alexa Fluor 555-conjugated goat anti-mouse IgG (Molecular Probes) as secondary antibodies. To detect induction of apoptosis in transfected RF/6A cells, nuclei of RF/6A cells were stained with 300 nM 4′,6-diamidino-2-phenylindole, dilactate (DAPI dilactate, Molecular Probes) for 5 min and then visualized under a fluorescence microscope. The permeabilized cells were incubated with the indicated primary antibodies in PGS solution for 1 h. The cells were washed with PBS three times to remove unbound antibody; bound antibodies were detected using the indicated dye-conjugated secondary antibodies.

For immunofluorescence labeling in *S. cerevisiae*, yeast cells were fixed in 4% paraformaldehyde solution for 1 h, and digested with lyticase (Sigma) at the final concentration of 100 U/ml for 30 min. Yeast cells were permeabilized with −20°C methanol for 5 min, and subjected to immunostaining with rabbit anti-Ats-1 antibody, and Alexa Fluor 488-conjugated goat anti-rabbit IgG.

Fluorescence images were observed using a Nikon Eclipse E400 fluorescence microscope with a xenon-mercury light source (Nikon Instruments, Melville, NY) or using an LSM 510 laser-scanning confocal microscope (Carl Zeiss, Thornwood, NY). A 3-D model was reconstructed with ZEN software (Zeiss) based on the Z-stack confocal slices. The original color emitted by excited Alexa Fluor 350 (blue), and DAPI (blue) was transformed to gray pseudocolor using Photoshop 7.0 software (Adobe, San Jose, CA).

Immunofluorescence microscopy of HeLa cells was performed as described previously [71].

### Transfection

HeLa cells were transfected with pEGFP-N1 plasmids containing Ats-1, Ats-1 (55–57), or pAts-1ΔN17 genes using Lipofectamine 2000 (Invitrogen) according to the manufacturer's protocol. RF/6A endothelial cells were transfected by electroporation as previously described [72]. Briefly, RF/6A cells were washed once with PBS and resuspended in RPMI-1640 without serum at a final cell density of 2×10^7^/ml. RF/6A cells (80 µl) were then mixed with 5 µg recombinant plasmid in a 0.2-cm cuvette and subjected to electroporation using the Gene Pulser Xcell System (Bio-Rad) at 100 V and 1000 µF.

### Induction of apoptosis and PARP cleavage

Transfected RF/6A cells (1.6×10^5^) growing on glass coverslips in 2 ml advanced MEM were treated with 100 µM etoposide (Sigma) for 1 day after 20 h post-transfection. Cells then were fixed with 2% paraformaldehyde for fluorescence microscopy to visualize cell morphology, nucleus condensation and fragmentation, and release of cytochrome *c* into the cytosol. To investigate the cleavage of PARP in apoptosis induction, RF/6A cells transfected with pAts-1, pAts-1 (55–57), or pGFP, were treated with 100 µM etoposide (Sigma) for 12 h after 20 h post-transfection, and subjected to Western blot analysis.

### Western blot analysis

For determining Ats-1, Mn-Sod, HA, actin, or Bax in *A. phagocytophilum*-infected HL-60 cells (2×10^6^, >95% infected), Percoll-purified *A. phagocytophilum* pellet, or transfected RF/6A cells (1.6×10^6^), cells were dissolved in 100 µl SDS-PAGE loading buffer, and boiled for 5 min. For detecting PARP cleavage, RF/6A cells (1.6×10^6^) were lysed in 200 µl sample buffer (6 M urea, 62.5 mM Tris-HCl, 10% glycerol, 5% β-mercaptoethanol, 2% SDS, 0.00125% bromophenol blue, pH 6.8), sonicated for 15 s, and incubated at 65°C for 15 min. Samples were separated by 10% SDS-PAGE, and transferred to a nitrocellulose membrane using a semi-dry blotter (WEP, Seattle, WA). The membrane was blocked using 5% (w/v) skim milk in TBS (pH 7.5), then incubated with rabbit anti-Ats-1 antibody (1∶1,000 dilution), mouse monoclonal anti-HA antibody (HA.11, Clone 16B12, Covance, Emeryville, CA, 1∶1,000 dilution), rabbit anti-Mn-Sod (Stressgen, 1∶2,000 dilution), rabbit anti-actin (Sigma, 1∶2,000 dilution), mouse monoclonal anti-Bax (1∶500), or rabbit anti-PARP (Cell Signal Technology, Danvers, MA, 1∶1,000 dilution) at 4°C for 12 h, and subsequently with a 1∶2,000 dilution of peroxidase-conjugated goat anti-rabbit IgG or peroxidase-conjugated goat anti-mouse IgG (KPL, Gaithersburg, MD) at RT for 3 h. Bound antibodies were detected using chemiluminescence (Pierce Biotechnology, Rockford, IL). For studying Ats-1 import into mitochondria, and sub-mitochondrial localization, Western blot analysis was performed using anti-Tim44 (BD Transduction laboratories), - Mitofilin (Abcam), -Tom40 (Santa Cruz Biotechnology), or -Hsp60 (Stressgen) antibodies. For studying Ats-1 expression and Bax translocation in yeast cells, Western blot analysis was performed using anti-Ats-1, -Bax, or - *S. cerevisiae* porin (Invitrogen). The images were captured using a CCD camera (Fuji LAS-3000 imaging system), and band density was measured by Fujifilm MultiGauge software.

### Expression of Ats-1, Ats-1(55–57), and Bax, and translocation of Bax to mitochondria in *Saccharomyces cerevisiae*

The DNA fragments encoding Ats-1 and Ats-1 (55–57) was amplified using primers Ats-1Y Forward and Ats-1Y Reverse (Table S1), and cloned into yeast constitutive expression vector pGADT7 AD (Clontech), resulting recombinant plasmids pYAts-1, and pYAts-1(55–57). DNA fragment encoding Bax was generated by using human neutrophil cDNA [20] as a template, and the primers Bax Forward and Bax Reverse (Table S1), and cloned into an inducible vector pYES2/ NT A (Invitrogen), resulting plasmid pBax. Three pairs of plasmids (pBax and pGADT7 AD, pBax and pYAts-1(55–57), pBax and pYAts-1) were co-transformed into *S. cerevisiae* haploid strain YPH499 (*MATa ade2-101 his3*-Δ*200 leu2*-Δ*1 lys2-801 trp1*-Δ*63 ura3-52*) (ATCC) by using YEASTMAKER Yeast Transformation System 2 (Clontech). Recombinant yeast cells were cultured in synthetic medium lacking uracil and leucine, and supplemented with 2% dextrose (SD medium) or 2% galactose (SG medium), specified elsewhere [73]. To determine the localization of Ats-1, and Ats-1 (55–57) in yeast cells, recombinant YPH499 cells were cultured in SG medium to 0.5 OD (A600), followed by incubation with 800 nM MitoTracker Red CMXRos (Invitrogen) for 30 min. Yeast cells were fixed and subjected to immunostaining with anti-Ats-1 antibody. To test whether Ats-1 could relieve the Bax-induced growth inhibition in yeast cells, recombinant YPH499 strains were cultured in liquid SD media overnight at 30°C, centrifuged and washed once with SG media, followed by dilution to 0.05 OD (A600) in SG media. Diluted recombinant YPH499 cells were cultured in SG media to induce Bax expression for five days. The viable yeast cells were determined at day 0 and day 5 by plate count technique.

To investigate Bax translocation to mitochondria in pBax and pGADT7 AD-, pBax and pYAts-1(55–57)-, or pBax and pYAts-1-co-transformed YPH499 cells, mitochondria were isolated from recombinant yeast cells after being cultured in Bax expression-inducing SG media for 12 h, according to described protocol [74]. Isolated mitochondria, along with yeast cell lysates, were subjected to Western blot, using anti-Ats-1, and anti- *S. cerevisiae* porin.

## Acknowledgments

The authors thank Dr. K. Green-Church at the Campus Chemical Instrument Center (CCIC) Mass Spectrometry and Proteomics Facility at Ohio State University for nano-LC/MS/MS.

## Supporting Information

Table S1 Primers used for PCR amplification of various *ats-1* constructs and *bax*.
(0.06 MB DOC)

Figure S1 HeLa cells were transfected with plasmids containing Ats-1, Ats-1 (55–57) or Ats-1 {capial Delta}N17. After 24 h of overexpression, cells were stained with mitochondrial membrane-potential sensitive dye MitoTracker Orange, fixed and analyzed by immunofluorescence, using antibodies against Ats-1 protein and Cy2 coupled secondary antibodies.
(1.31 MB TIF)

Figure S2 Yeast cells co-transformed with pBax, and pGADT7 AD (GADT7), pYAts-1(55–57), or pYAts-1, were cultured in galactose medium for 12 h to induce Bax expression. Total cell lysate, and isolated mitochondria were subjected to SDS-PAGE, and transferred to nitrocellulose membrane. Transferred membrane was stained by ponceau S to compare the total protein loading amount among samples. For isolated mitochondria group, or total lysate group, there are no differences in loading amount among GADT7, Ats-1(55–57), and Ats-1 lane.
(0.21 MB TIF)

Video S1 Movie for 3-D reconstruction showing Ats-1 localization in *A. phagocytophilum*-infected HL-60 cells.
(0.78 MB MOV)

## References

[1] Bakken JS, Dumler S (2008). Human granulocytic anaplasmosis.. Infect Dis Clin North Am.

[2] Thomas RJ, Dumler JS, Carlyon JA (2009). Current management of human granulocytic anaplasmosis, human monocytic ehrlichiosis and *Ehrlichia ewingii* ehrlichiosis.. Expert Rev Anti Infect Ther.

[3] Rikihisa Y (2009). Molecular events involved in cellular invasion by *Ehrlichia chaffeensis* and *Anaplasma phagocytophilum*.. Vet Parasitol.

[4] Dunning Hotopp JC, Lin M, Madupu R, Crabtree J, Angiuoli SV (2006). Comparative genomics of emerging human ehrlichiosis agents.. PLoS Genet.

[5] Rikihisa Y (2006). *Ehrlichia* subversion of host innate responses.. Curr Opin Microbiol.

[6] Labbe K, Saleh M (2008). Cell death in the host response to infection.. Cell Death Differ.

[7] Akgul C, Moulding DA, Edwards SW (2001). Molecular control of neutrophil apoptosis.. FEBS Lett.

[8] Yoshiie K, Kim HY, Mott J, Rikihisa Y (2000). Intracellular infection by the human granulocytic ehrlichiosis agent inhibits human neutrophil apoptosis.. Infect Immun.

[9] Scaife H, Woldehiwet Z, Hart CA, Edwards SW (2003). *Anaplasma phagocytophilum* reduces neutrophil apoptosis in vivo.. Infect Immun.

[10] Borjesson DL, Kobayashi SD, Whitney AR, Voyich JM, Argue CM (2005). Insights into pathogen immune evasion mechanisms: *Anaplasma phagocytophilum* fails to induce an apoptosis differentiation program in human neutrophils.. J Immunol.

[11] Choi KS, Park JT, Dumler JS (2005). *Anaplasma phagocytophilum* delay of neutrophil apoptosis through the p38 mitogen-activated protein kinase signal pathway.. Infect Immun.

[12] Ge Y, Yoshiie K, Kuribayashi F, Lin M, Rikihisa Y (2005). *Anaplasma phagocytophilum* inhibits human neutrophil apoptosis via upregulation of *bfl-1*, maintenance of mitochondrial membrane potential and prevention of caspase 3 activation.. Cell Microbiol.

[13] Ge Y, Rikihisa Y (2006). *Anaplasma phagocytophilum* delays spontaneous human neutrophil apoptosis by modulation of multiple apoptotic pathways.. Cell Microbiol.

[14] Lee HC, Goodman JL (2006). *Anaplasma phagocytophilum* causes global induction of antiapoptosis in human neutrophils.. Genomics.

[15] Alvarez-Martinez CE, Christie PJ (2009). Biological Diversity of Prokaryotic Type IV Secretion Systems.. Microbiol Mol Biol Rev.

[16] Laguna RK, Creasey EA, Li Z, Valtz N, Isberg RR (2006). A *Legionella pneumophila*-translocated substrate that is required for growth within macrophages and protection from host cell death.. Proc Natl Acad Sci U S A.

[17] Schmid MC, Scheidegger F, Dehio M, Balmelle-Devaux N, Schulein R (2006). A translocated bacterial protein protects vascular endothelial cells from apoptosis.. PLoS Pathog.

[18] Banga S, Gao P, Shen X, Fiscus V, Zong WX (2007). *Legionella pneumophila* inhibits macrophage apoptosis by targeting pro-death members of the Bcl2 protein family.. Proc Natl Acad Sci U S A.

[19] Ohashi N, Zhi N, Lin Q, Rikihisa Y (2002). Characterization and transcriptional analysis of gene clusters for a type IV secretion machinery in human granulocytic and monocytic ehrlichiosis agents.. Infect Immun.

[20] Niu H, Rikihisa Y, Yamaguchi M, Ohashi N (2006). Differential expression of VirB9 and VirB6 during the life cycle of *Anaplasma phagocytophilum* in human leucocytes is associated with differential binding and avoidance of lysosome pathway.. Cell Microbiol.

[21] Lin M, den Dulk-Ras A, Hooykaas PJ, Rikihisa Y (2007). *Anaplasma phagocytophilum* AnkA secreted by type IV secretion system is tyrosine phosphorylated by Abl-1 to facilitate infection.. Cell Microbiol.

[22] Cascales E, Christie PJ (2003). The versatile bacterial type IV secretion systems.. Nat Rev Microbiol.

[23] Vergunst AC, van Lier MC, den Dulk-Ras A, Stuve TA, Ouwehand A (2005). Positive charge is an important feature of the C-terminal transport signal of the VirB/D4-translocated proteins of *Agrobacterium*.. Proc Natl Acad Sci U S A.

[24] Cascales E, Christie PJ (2004). *Agrobacterium* VirB10, an ATP energy sensor required for type IV secretion.. Proc Natl Acad Sci U S A.

[25] Atmakuri K, Cascales E, Christie PJ (2004). Energetic components VirD4, VirB11 and VirB4 mediate early DNA transfer reactions required for bacterial type IV secretion.. Mol Microbiol.

[26] Cascales E, Christie PJ (2004). Definition of a bacterial type IV secretion pathway for a DNA substrate.. Science.

[27] Zhi N, Ohashi N, Rikihisa Y, Horowitz HW, Wormser GP (1998). Cloning and expression of the 44-kilodalton major outer membrane protein gene of the human granulocytic ehrlichiosis agent and application of the recombinant protein to serodiagnosis.. J Clin Microbiol.

[28] Kim HY, Rikihisa Y (1998). Characterization of monoclonal antibodies to the 44-kilodalton major outer membrane protein of the human granulocytic ehrlichiosis agent.. J Clin Microbiol.

[29] Claros MG, Vincens P (1996). Computational method to predict mitochondrially imported proteins and their targeting sequences.. Eur J Biochem.

[30] Small I, Peeters N, Legeai F, Lurin C (2004). Predotar: A tool for rapidly screening proteomes for N-terminal targeting sequences.. Proteomics.

[31] Munderloh UG, Lynch MJ, Herron MJ, Palmer AT, Kurtti TJ (2004). Infection of endothelial cells with *Anaplasma marginale* and *A. phagocytophilum*.. Vet Microbiol.

[32] Herron MJ, Ericson ME, Kurtti TJ, Munderloh UG (2005). The interactions of *Anaplasma phagocytophilum*, endothelial cells, and human neutrophils.. Ann N Y Acad Sci.

[33] Omura T (1998). Mitochondria-targeting sequence, a multi-role sorting sequence recognized at all steps of protein import into mitochondria.. J Biochem.

[34] Milenkovic D, Muller J, Stojanovski D, Pfanner N, Chacinska A (2007). Diverse mechanisms and machineries for import of mitochondrial proteins.. Biol Chem.

[35] Karpinich NO, Tafani M, Rothman RJ, Russo MA, Farber JL (2002). The course of etoposide-induced apoptosis from damage to DNA and p53 activation to mitochondrial release of cytochrome c.. J Biol Chem.

[36] Willis SN, Chen L, Dewson G, Wei A, Naik E (2005). Proapoptotic Bak is sequestered by Mcl-1 and Bcl-xL, but not Bcl-2, until displaced by BH3-only proteins.. Genes Dev.

[37] Nechushtan A, Smith CL, Lamensdorf I, Yoon SH, Youle RJ (2001). Bax and Bak coalesce into novel mitochondria-associated clusters during apoptosis.. J Cell Biol.

[38] Antonsson B, Montessuit S, Lauper S, Eskes R, Martinou JC (2000). Bax oligomerization is required for channel-forming activity in liposomes and to trigger cytochrome *c* release from mitochondria.. Biochem J.

[39] Lartigue L, Medina C, Schembri L, Chabert P, Zanese M (2008). An intracellular wave of cytochrome *c* propagates and precedes Bax redistribution during apoptosis.. J Cell Sci.

[40] Kroemer G, Reed JC (2000). Mitochondrial control of cell death.. Nat Med.

[41] Satoh MS, Lindahl T (1992). Role of poly(ADP-ribose) formation in DNA repair.. Nature.

[42] Nicholson DW, Ali A, Thornberry NA, Vaillancourt JP, Ding CK (1995). Identification and inhibition of the ICE/CED-3 protease necessary for mammalian apoptosis.. Nature.

[43] Casiano CA, Martin SJ, Green DR, Tan EM (1996). Selective cleavage of nuclear autoantigens during CD95 (Fas/APO-1)-mediated T cell apoptosis.. J Exp Med.

[44] Ligr M, Madeo F, Frohlich E, Hilt W, Frohlich KU (1998). Mammalian Bax triggers apoptotic changes in yeast.. FEBS Lett.

[45] Priault M, Camougrand N, Kinnally KW, Vallette FM, Manon S (2003). Yeast as a tool to study Bax/mitochondrial interactions in cell death.. FEMS Yeast Res.

[46] Burstein D, Zusman T, Degtyar E, Viner R, Segal G (2009). Genome-scale identification of *Legionella pneumophila* effectors using a machine learning approach.. PLoS Pathog.

[47] Voth DE, Howe D, Beare PA, Vogel JP, Unsworth N (2009). The *Coxiella burnetii* ankyrin repeat domain-containing protein family is heterogeneous, with C-terminal truncations that influence Dot/Icm-mediated secretion.. J Bacteriol.

[48] Schulein R, Guye P, Rhomberg TA, Schmid MC, Schroder G (2005). A bipartite signal mediates the transfer of type IV secretion substrates of *Bartonella henselae* into human cells.. Proc Natl Acad Sci U S A.

[49] de Jong MF, Sun YH, den Hartigh AB, van Dijl JM, Tsolis RM (2008). Identification of VceA and VceC, two members of the VjbR regulon that are translocated into macrophages by the *Brucella* type IV secretion system.. Mol Microbiol.

[50] Hendrick JP, Hodges PE, Rosenberg LE (1989). Survey of amino-terminal proteolytic cleavage sites in mitochondrial precursor proteins: leader peptides cleaved by two matrix proteases share a three-amino acid motif.. Proc Natl Acad Sci U S A.

[51] Kozjak-Pavlovic V, Ross K, Benlasfer N, Kimmig S, Karlas A (2007). Conserved roles of Sam50 and metaxins in VDAC biogenesis.. EMBO Rep.

[52] Derre I, Pypaert M, Dautry-Varsat A, Agaisse H (2007). RNAi screen in Drosophila cells reveals the involvement of the Tom complex in *Chlamydia* infection.. PLoS Pathog.

[53] Nougayrede JP, Donnenberg MS (2004). Enteropathogenic *Escherichia coli* EspF is targeted to mitochondria and is required to initiate the mitochondrial death pathway.. Cell Microbiol.

[54] Munkvold KR, Martin ME, Bronstein PA, Collmer A (2008). A survey of the *Pseudomonas syringae* pv. *tomato* DC3000 type III secretion system effector repertoire reveals several effectors that are deleterious when expressed in *Saccharomyces cerevisiae*.. Mol Plant Microbe Interact.

[55] Layton AN, Brown PJ, Galyov EE (2005). The *Salmonella* translocated effector SopA is targeted to the mitochondria of infected cells.. J Bacteriol.

[56] Degtyar E, Zusman T, Ehrlich M, Segal G (2009). A *Legionella effector* acquired from protozoa is involved in sphingolipids metabolism and is targeted to the host cell mitochondria.. Cell Microbiol.

[57] Faherty CS, Maurelli AT (2008). Staying alive: bacterial inhibition of apoptosis during infection.. Trends Microbiol.

[58] Pirbhai M, Dong F, Zhong Y, Pan KZ, Zhong G (2006). The secreted protease factor CPAF is responsible for degrading pro-apoptotic BH3-only proteins in *Chlamydia trachomatis*-infected cells.. J Biol Chem.

[59] Verbeke P, Welter-Stahl L, Ying S, Hansen J, Hacker G (2006). Recruitment of BAD by the *Chlamydia trachomatis* vacuole correlates with host-cell survival.. PLoS Pathog.

[60] Rajalingam K, Sharma M, Paland N, Hurwitz R, Thieck O (2006). IAP-IAP complexes required for apoptosis resistance of *C. trachomatis*-infected cells.. PLoS Pathog.

[61] Rajalingam K, Sharma M, Lohmann C, Oswald M, Thieck O (2008). Mcl-1 is a key regulator of apoptosis resistance in *Chlamydia trachomatis*-infected cells.. PLoS ONE.

[62] Massari P, Ho Y, Wetzler LM (2000). *Neisseria meningitidis* porin PorB interacts with mitochondria and protects cells from apoptosis.. Proc Natl Acad Sci U S A.

[63] Scorrano L, Ashiya M, Buttle K, Weiler S, Oakes SA (2002). A distinct pathway remodels mitochondrial cristae and mobilizes cytochrome *c* during apoptosis.. Dev Cell.

[64] McCormick AL, Smith VL, Chow D, Mocarski ES (2003). Disruption of mitochondrial networks by the human cytomegalovirus UL37 gene product viral mitochondrion-localized inhibitor of apoptosis.. J Virol.

[65] Smaili SS, Hsu YT, Sanders KM, Russell JT, Youle RJ (2001). Bax translocation to mitochondria subsequent to a rapid loss of mitochondrial membrane potential.. Cell Death Differ.

[66] Sawai H, Domae N (2008). Release of cytochrome *c* from mitochondria precedes Bax translocation/activation in Triton X-100-induced apoptosis.. Leuk Res.

[67] Lucken-Ardjomande S, Montessuit S, Martinou JC (2008). Contributions to Bax insertion and oligomerization of lipids of the mitochondrial outer membrane.. Cell Death Differ.

[68] Forte M, Bernardi P (2006). The permeability transition and BCL-2 family proteins in apoptosis: co-conspirators or independent agents?. Cell Death Differ.

[69] Brdiczka DG, Zorov DB, Sheu SS (2006). Mitochondrial contact sites: their role in energy metabolism and apoptosis.. Biochim Biophys Acta.

[70] Lin M, Rikihisa Y (2003). *Ehrlichia chaffeensis* and *Anaplasma phagocytophilum* lack genes for lipid A biosynthesis and incorporate cholesterol for their survival.. Infect Immun.

[71] Muller A, Rassow J, Grimm J, Machuy N, Meyer TF (2002). VDAC and the bacterial porin PorB of *Neisseria gonorrhoeae* share mitochondrial import pathways.. Embo J.

[72] Niu H, Yamaguchi M, Rikihisa Y (2008). Subversion of cellular autophagy by A*naplasma phagocytophilum*.. Cell Microbiol.

[73] Amberg DC, Daniel J, Burke DJ, Strathern JN (2005). *Methods in Yeast Genetics*: A Cold Spring Harbor Laboratory Course Manual..

[74] Xiao W (2006). Yeast protocols..

