# Supplementary material for: Anaplasma phagocytophilum Ats-1 Is Imported into Host Cell Mitochondria and Interferes with Apoptosis Induction
Source: PLoS Pathog. 2010 Feb 19;6(2):e1000774. doi: 10.1371/journal.ppat.1000774 (PMC2824752; doi:10.1371/journal.ppat.1000774)
Supplement: Table S1 — Primers used for PCR amplification of various ats-1 constructs and bax. (0.06 MB DOC) [file ppat.1000774.s001.doc]

**Table S1.** Primers used for PCR amplification of various *ats-1* constructs and *bax*.

| Primer ID | Sequence | Note |
| --- | --- | --- |
| APH0859 Expression Forward | 5’-GTGCGGCCGCAATGGATATTGGCGCCAGAATG-3’ | NotI site is underlined |
| APH0859 Expression Reverse | 5’-TTACTCGAGTTACCTCGTACCTTTACCATGTG-3’ | XhoI site is underlined |
| Ats-1 Forward | 5′-GCAGTCGACGCCACCATGGTGCTAATAAGAAGAATTCTGAC-3′ | Sal I site is underlined |
| Ats-1 Reverse | 5′-AGTGCGGCCGCTTACCTCGTACCTTTACCATGTG-3′ | Not I site is underlined |
| 30–31 HA Reverse N | 5′-*CATCGTATGGGTA*GCTAGTATTTTCAGTGGCTGG-3′ | Sequence encoding HA tag is italicized |
| 30–31 HA Forward C1 | 5′-*CCAGATTACGCT*GCGAGAACCTCAAGAAATCTC-3′ | Sequence encoding HA tag is italicized |
| 30–31 HA Forward C2 | 5′-*TACCCATACGATGTTCCAGATTACGCT*GCGAGAACC-3′ | Sequence encoding HA tag is italicized |
| 30–31 HA Forward C3 | 5′-CTGAAAATACTAGC*TACCCATACGATGTTCCAGAT*-3′ | Sequence encoding HA tag is italicized |
| 45–46 HA Reverse N | 5′-*CATCGTATGGGTA*AAAGAAATTTCCTGTTGTACC-3′ | Sequence encoding HA tag is italicized |
| 45–46 HA Forward C1 | 5′-*CCAGATTACGCT*AATGGCCTCATGGGTAAAGGA-3′ | Sequence encoding HA tag is italicized |
| 45–46 HA Forward C2 | 5′-*TACCCATACGATGTTCCAGATTACGCT*AATGGCCTC-3′ | Sequence encoding HA tag is italicized |
| 45–46 HA Forward C3 | 5′-CAGGAAATTTCTTT*TACCCATACGATGTTCCAGAT*-3′ | Sequence encoding HA tag is italicized |
| 60–61 HA Reverse N | 5′-*CATCGTATGGGTA*AGAAGCGCGATGGTAAAAAGG-3′ | Sequence encoding HA tag is italicized |
| 60–61 HA Forward C1 | 5′-*CCAGATTACGCT*GAGATGCAGAATCTCCCCTGG-3′ | Sequence encoding HA tag is italicized |
| 60–61 HA Forward C2 | 5′-*TACCCATACGATGTTCCAGATTACGCT*GAGATGCAG-3′ | Sequence encoding HA tag is italicized |
| 60–61 HA Forward C3 | 5′-ACCATCGCGCTTCT*TACCCATACGATGTTCCAGAT*-3′ | Sequence encoding HA tag is italicized |
| 72–73 HA Reverse N | 5′-*CGTATGGGTA*GCCCCTCTCTTTATCCCAGG-3′ | Sequence encoding HA tag is italicized |
| 72–73 HA Forward C1 | 5′-*CCAGATTACGCT*ACAAAAATAAGTTCGCACTATGC-3′ | Sequence encoding HA tag is italicized |
| 72–73 HA Forward C2 | 5′-*TACCCATACGATGTTCCAGATTACGCT*ACAAAAATA-3′ | Sequence encoding HA tag is italicized |
| 72–73 HA Forward C3 | 5′-AAGAGAGGGGC*TACCCATACGATGTTCCAGA*-3′ | Sequence encoding HA tag is italicized |
| 46–48 AAA Forward | 5′-TTCTTTGCTGCTGCTATGGGTAAAGGAAAGCCTTTTTA-3′ | Sequence encoding amino acid substitution is underlined |
| 46–48 AAA Reverse | 5′-ACCCATAGCAGCAGCAAAGAAATTTCCTGTTGTACC-3′ | Sequence encoding amino acid substitutions are underlined |
| 49 – 51 AAA Forward | 5′-GGCCTCGCTGCTGCTGGAAAGCCTTTTTACCATCGC-3′ | Sequence encoding amino acid substitutions are underlined |
| 49–51 AAA Reverse | 5′-CTTTCCAGCAGCAGCGAGGCCATTAAAGAAATTTCC-3′ | Sequence for amino acid substitution is underlined |
| 52 – 54 AAA Forward | 5′-GGTAAAGCTGCTGCTTTTTACCATCGCGCTTCTGAG-3′ | Sequence encoding amino acid substitutions are underlined |
| 52–54 AAA Reverse | 5′-GTAAAAAGCAGCAGCTTTACCCATGAGGCCATTAAAG-3′ | Sequence encoding amino acid substitutions are underlined |
| 55–57 AAA Forward | 5′- AAGCCTGCTGCTGCTCGCGCTTCTGAGATGCAGAAT-3′ | Sequence encoding amino acid substitutions are underlined |
| 55–57 AAA Reverse | 5′-AGCGCGAGCAGCAGCAGGCTTTCCTTTACCCATGAG-3′ | Sequence encoding amino acid substitutions are underlined |
| 58–60 AGA Forward | 5′-TACCATGCTGGCGCTGAGATGCAGAATCTCCCCTGG-3′ | Sequence encoding amino acid substitutions are underlined |
| 58–60 AGA Reverse | 5′-CATCTCAGCGCCAGCATGGTAAAAAGGCTTTCCTTTAC-3′ | Sequence encoding amino acid substitutions are underlined |
| Ats-1 delta N17 Forward | 5′-GCAGTCGACGCCACCATGGCGCGCATTGTTTCTGGATTC-3′ | Sal I site is underlined |
| Ats-1 Free N Forward | 5’-ATACATATGctaataagaagaattctg-3’ | Nde I site is underlined |
| Ats-1 Free N delta 17 Forward | 5’-ATACATATGgtttctggattcacagctcca-3’ | Nde I site is underlined |
| Ats-1 Free N Reverse | 5’-GTGCTCGAGcctcgtacctttaccatgtgt-3’ | Xho I site is underlined |
| Ats-1Y Forward | 5’-CCAAGCTTTGCAAAGatgctaataagaagaattctg-3’ | Hind III site is underlined |
| Ats-1Y Reverse | 5’-CCAAGCTTTTACCTCGTACCTTTACCATG-3’ | Hind III site is underlined |
| Bax Forward | 5’-ATTAAGCTTACC atggacgggtccggggagcagc-3’ | Hind III site is underlined |
| Bax Reverse | 5’- agtgcggccgctcagcccatcttcttccagatg-3’ | Not I site is underlined |
