# Supplementary figures and images for: Anaplasma phagocytophilum Ats-1 Is Imported into Host Cell Mitochondria and Interferes with Apoptosis Induction
Source: PLoS Pathog. 2010 Feb 19;6(2):e1000774. doi: 10.1371/journal.ppat.1000774 (PMC2824752; doi:10.1371/journal.ppat.1000774)

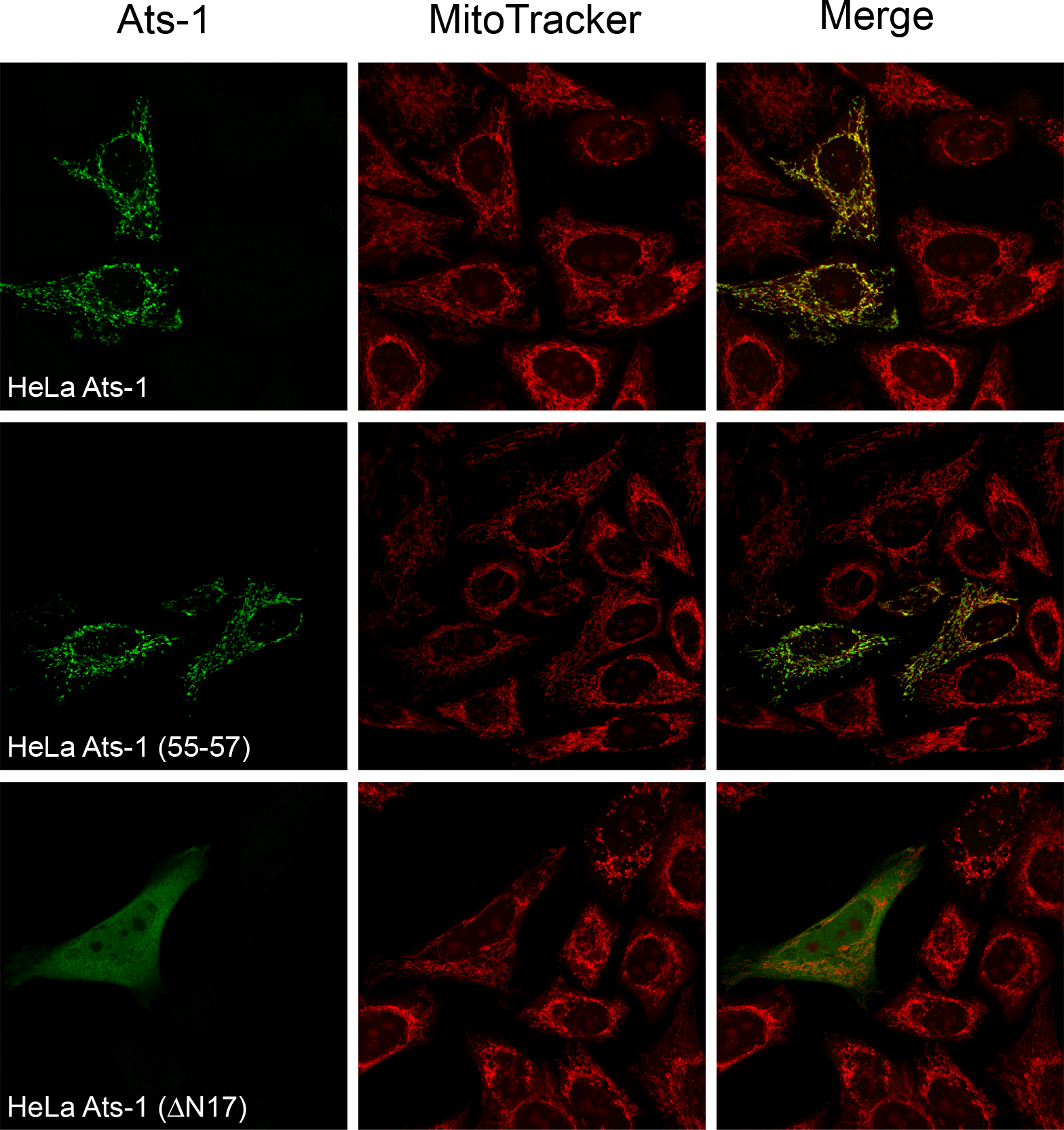

Supplement: Figure S1 — HeLa cells were transfected with plasmids containing Ats-1, Ats-1 (55–57) or Ats-1 {capial Delta}N17. After 24 h of overexpression, cells were stained with mitochondrial membrane-potential sensitive dye MitoTracker Orange, fixed and analyzed by immunofluorescence, using antibodies against Ats-1 protein and Cy2 coupled secondary antibodies. (1.31 MB TIF) [file ppat.1000774.s002.tif]

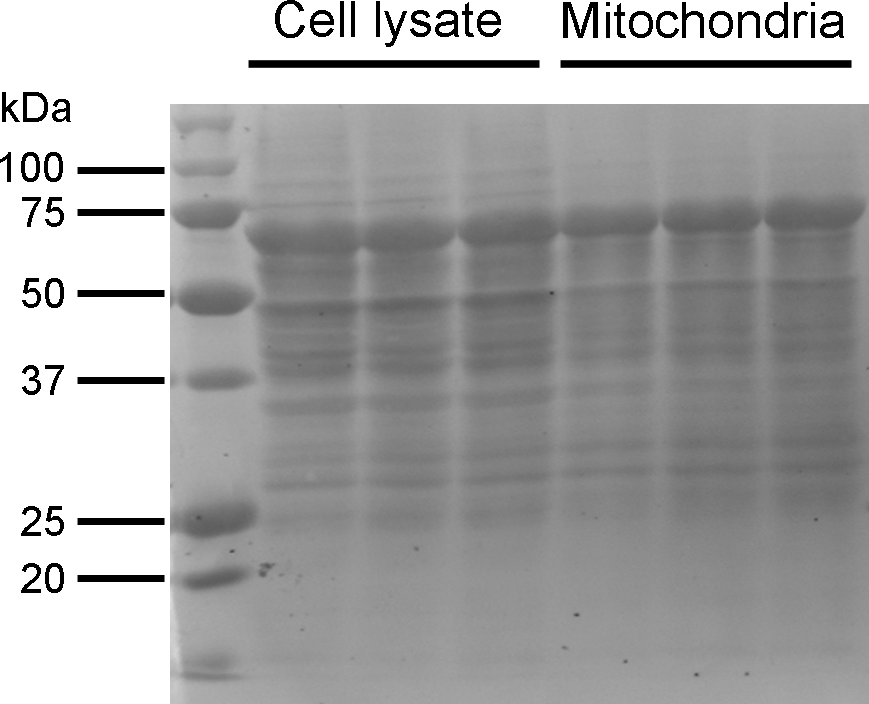

Supplement: Figure S2 — Yeast cells co-transformed with pBax, and pGADT7 AD (GADT7), pYAts-1(55–57), or pYAts-1, were cultured in galactose medium for 12 h to induce Bax expression. Total cell lysate, and isolated mitochondria were subjected to SDS-PAGE, and transferred to nitrocellulose membrane. Transferred membrane was stained by ponceau S to compare the total protein loading amount among samples. For isolated mitochondria group, or total lysate group, there are no differences in loading amount among GADT7, Ats-1(55–57), and Ats-1 lane. (0.21 MB TIF) [file ppat.1000774.s003.tif]
